# *In silico* metabolic network analysis of *Arabidopsis* leaves

**DOI:** 10.1186/s12918-016-0347-3

**Published:** 2016-10-29

**Authors:** Veronique Beckers, Lisa Maria Dersch, Katrin Lotz, Guido Melzer, Oliver Ernst Bläsing, Regine Fuchs, Thomas Ehrhardt, Christoph Wittmann

**Affiliations:** 1 Institute for Systems Biotechnology, Saarland University, Campus A1.5, 66123 Saarbrücken, Germany; 2 Metanomics GmbH, Berlin, Germany; 3 Institute of Biochemical Engineering, Technical University Braunschweig, Braunschweig, Germany

**Keywords:** Elementary flux modes, Arabidopsis, Day-night shift, In vivo, In silico, Electron flow, Transcriptome, Fluxome

## Abstract

**Background:**

During the last decades, we face an increasing interest in superior plants to supply growing demands for human and animal nutrition and for the developing bio-based economy. Presently, our limited understanding of their metabolism and its regulation hampers the targeted development of desired plant phenotypes. In this regard, systems biology, in particular the integration of metabolic and regulatory networks, is promising to broaden our knowledge and to further explore the biotechnological potential of plants.

**Results:**

The thale cress *Arabidopsis thaliana* provides an ideal model to understand plant primary metabolism. To obtain insight into its functional properties, we constructed a large-scale metabolic network of the leaf of *A. thaliana*. It represented 511 reactions with spatial separation into compartments. Systematic analysis of this network, utilizing elementary flux modes, investigates metabolic capabilities of the plant and predicts relevant properties on the systems level: optimum pathway use for maximum growth and flux re-arrangement in response to environmental perturbation. Our computational model indicates that the *A. thaliana* leaf operates near its theoretical optimum flux state in the light, however, only in a narrow range of photon usage. The simulations further demonstrate that the natural day-night shift requires substantial re-arrangement of pathway flux between compartments: 89 reactions, involving redox and energy metabolism, substantially change the extent of flux, whereas 19 reactions even invert flux direction. The optimum set of anabolic pathways differs between day and night and is partly shifted between compartments. The integration with experimental transcriptome data pinpoints selected transcriptional changes that mediate the diurnal adaptation of the plant and superimpose the flux response.

**Conclusions:**

The successful application of predictive modelling in *Arabidopsis thaliana* can bring systems-biological interpretation of plant systems forward. Using the gained knowledge, metabolic engineering strategies to engage plants as biotechnological factories can be developed.

**Electronic supplementary material:**

The online version of this article (doi:10.1186/s12918-016-0347-3) contains supplementary material, which is available to authorized users.

## Background

Increasing world population, shortage of arable land and the resulting growing demand for food, feed and raw materials are major drivers to create plant lines with increased performance, e.g. better resistance to disease and drought [1]. In addition, plants play a significant role for the developing bio-economy [2] and emerge as platforms for sustainable production of therapeutics, renewable chemicals and biofuels, purely from sunlight and carbon dioxide [1], which adds impetus to the growing interest in enhanced crops [3, 4]. Admittedly, plant metabolic engineering and plant systems metabolic engineering is hampered by our still limited understanding of the underlying metabolism and its regulation [5, 6], involving only little systems-level understanding of the effect of genetic modifications [7, 8]. It is therefore easy to understand that the interest in methods to drive plant metabolic engineering is high [6, 7, 9]. One promising approach to support plant metabolic engineers, obviously, is the use of *in silico* metabolic modelling. Firstly, *in silico* metabolic modelling has proven impressively successful in guiding systems metabolic engineering of other biological systems, including bacteria and fungi [10–14]. Secondly, the over 50, fully sequenced plant genomes, mainly crops, [15] provide a valuable source of information to create plant metabolic models [16–18]. And thirdly, a powerful collection of modelling approaches, currently available to model and simulate stoichiometric metabolic networks, can be adapted to plant metabolic networks in a straightforward manner [19]. Among the available modelling approaches are *in silico* based analyses, such as elementary flux mode analysis [20, 21] and extreme pathway analysis [22, 23], as well as analyses, which rely on experimental data to deliver necessary constraints, such as ^13^C-metabolic flux analysis (^13^C-MFA) [24–26], high-throughput isotope based metabolic screening [27], flux balance analysis [16] and metabolic control analysis [28]. Among the approaches, *in silico* simulation appears particularly interesting due to its high speed, given e.g. the relatively long time of experiments with growing plants.

Here, we conduct detailed *in silico* analysis of plant central carbon metabolism. We use *A. thaliana* as a widely applied model plant to study plant biology and biotechnology, reflected also by the fact that it was the first plant sequenced [29]. In short, a metabolic network model of the *A. thaliana* leaf was constructed, which represented 511 reactions and 1567 metabolic genes. Its reactions and pathways were localized in different subcellular compartments: cytosol, plastid, mitochondrion and peroxisome. Through simulation, relevant physiological scenarios of plant metabolism were studied. This involved the detailed investigation of the diurnal metabolism through comparison of metabolic capabilities in the light and in the dark. In addition, the modelling results were integrated with previous experimental fluxomic and transcriptomic data of *A. thaliana* leaves in order to explore the systems-wide regulation of the metabolism. This provides an enhanced understanding of plant metabolic pathways and their contribution to growth and product formation.

## Methods

### Metabolic network construction

The metabolic network used in this study comprises the central carbon metabolism of *A. thaliana* leaves. For re-construction, the initial draft of the network built on recent genome-scale models of the organism [30–32]. The derived pathway bibliography was then carefully checked and updated with recent findings, collected in metabolic pathway databases: Kyoto Encyclopedia of Genes and Genomes [33] (http://www.genome.jp/kegg), MetaCrop [34] (http://metacrop.ipk-gatersleben.de/) and AraCyc [35] (http://pmn.plantcyc.org). This provided state-of-art gross information on the genomic pathway repertoire. Where needed, the network was updated with experimental data and primary literature as described in detail below. Individual additions and specifications considered enzyme localization, cofactor usage, and inter-compartmental transport. Subsequently, the model was condensed, which, however, did not reduce its information content (Additional file 1: Information S1). Furthermore, the applied toolbox for the enumeration of elementary flux modes required *in silico* external exchange reactions for unbalanced metabolites (biomass, ATP for cellular maintenance, inorganic phosphate, photons and starch), which were added. Both models, the detailed version and the condensed version, used for the simulations, are provided as SBML files in the Additional files 2 and 3.

### Computation of elementary flux modes

Elementary flux modes (EFMs) were calculated with efmtool, based on the null space approach and recursive enumeration with bit pattern trees [21]. The EFM matrix, computed by the algorithm, comprises information on all thermodynamically and stoichiometrically possible pathways in the cell, which reduce metabolism into all feasible, unique, non-decomposable biochemical pathways [20]. All simulations were conducted on a quad core personal computer. Normalization of the EFM matrix and subsequent data interpretation was conducted as described previously [36]. In short, relative fluxes were normalized to their respective substrate uptake and the theoretical biomass production of each elementary flux mode was determined. The respective flux modes were expressed in (C-mol) (C-mol)^−1^. The relative contribution of a particular pathway to anabolic precursor formation was obtained by dividing the underlying pathway flux by the sum of all fluxes, which formed this anabolic precursor. Significant changes between two conditions were identified on the basis of a two-sample *t*-test (95 % significance level, p-value <0.05) and an absolute log2-value > 0.5.

### Transcriptome data processing

Experimental transcriptome data were taken from a recent study on the shift of gene expression between day and night in *A. thaliana* rosettes [37]. In this previous work, the amplitude limit of gene expression (log2 value) during the diurnal cycle was quantified by ATH1 arrays, and changes in absolute gene expression level were identified with a cut off value of 0.8. From the published data set, we extracted the genes encoding proteins of the central carbon metabolism. The obtained raw data were then inspected to identify the genes, which exhibited a diurnal expression change, i.e. revealed an unambiguous increase or decrease in expression during illumination and the opposite change during the dark period.

### Fluxome data processing

The experimentally measured metabolic fluxes of an illuminated *A. thaliana* rosette [38] were converted into (C-mol) (C-mol)^−1^ to enable a straightforward comparison with the respective flux modes, obtained in this work, also given in (C-mol) (C-mol)^−1^.

### Quantum yield data processing

The quantum yield of photosynthesis was derived from previous measurement of the rate of photosynthesis of *Arabidopsis* and of other C_3_ plants under ambient atmospheric conditions at different light intensity [39–43]. Due to the fact, that not all harvested quanta are converted into chemical energy, as some are lost through absorption by pigments, unable to contribute their excitation energy to photosynthesis, the experimental quantum yield values were corrected, assuming that 47 % of photons are outside the photosynthetically active range [44]. This provided a direct correlation between assimilated carbon dioxide and properly absorbed photons.

## Results

### Metabolic network topology

**Fig. 1:**
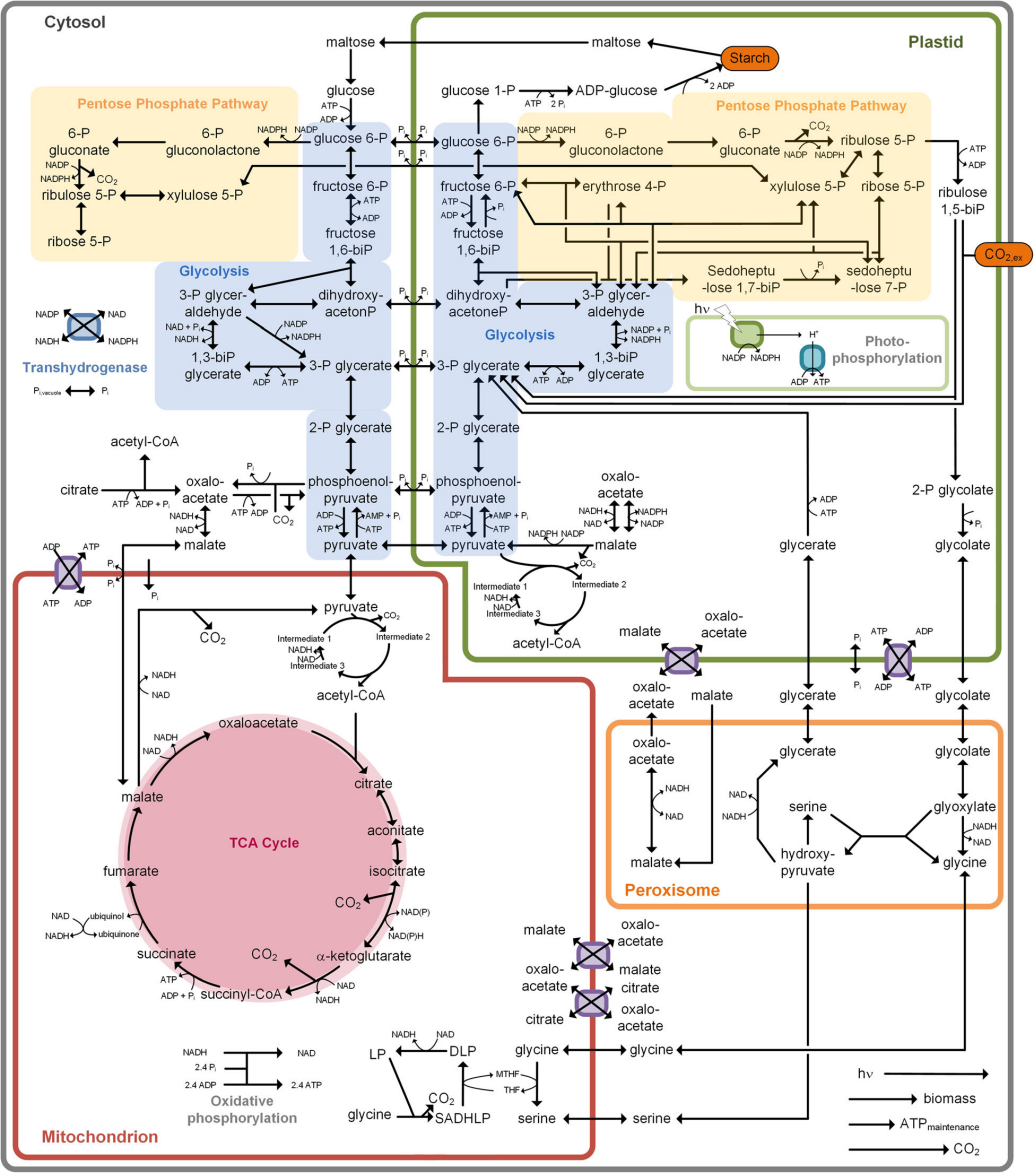
Condensed representation of the compartmented metabolic network of *A. thaliana*. Metabolic network for *Arabidopsis* leaf showing the carbon core metabolic pathways across four compartments: cytosol, plastid, mitochondrion and peroxisome. The purple transmembrane transporters mediate antiport of selected metabolites. Unidirectional and bidirectional transport is represented by unidirectional and bidirectional arrows across the membrane, respectively. CO_2_ is allowed to freely diffuse within the cell, whereas photons can pass both the cytosolic and the plastid membrane. A more detailed view on the system of photophosphorylation is provided in Fig. 2. For visualization purposes, anabolism is shown in a condensed form as single biomass forming reaction

**Fig. 2:**
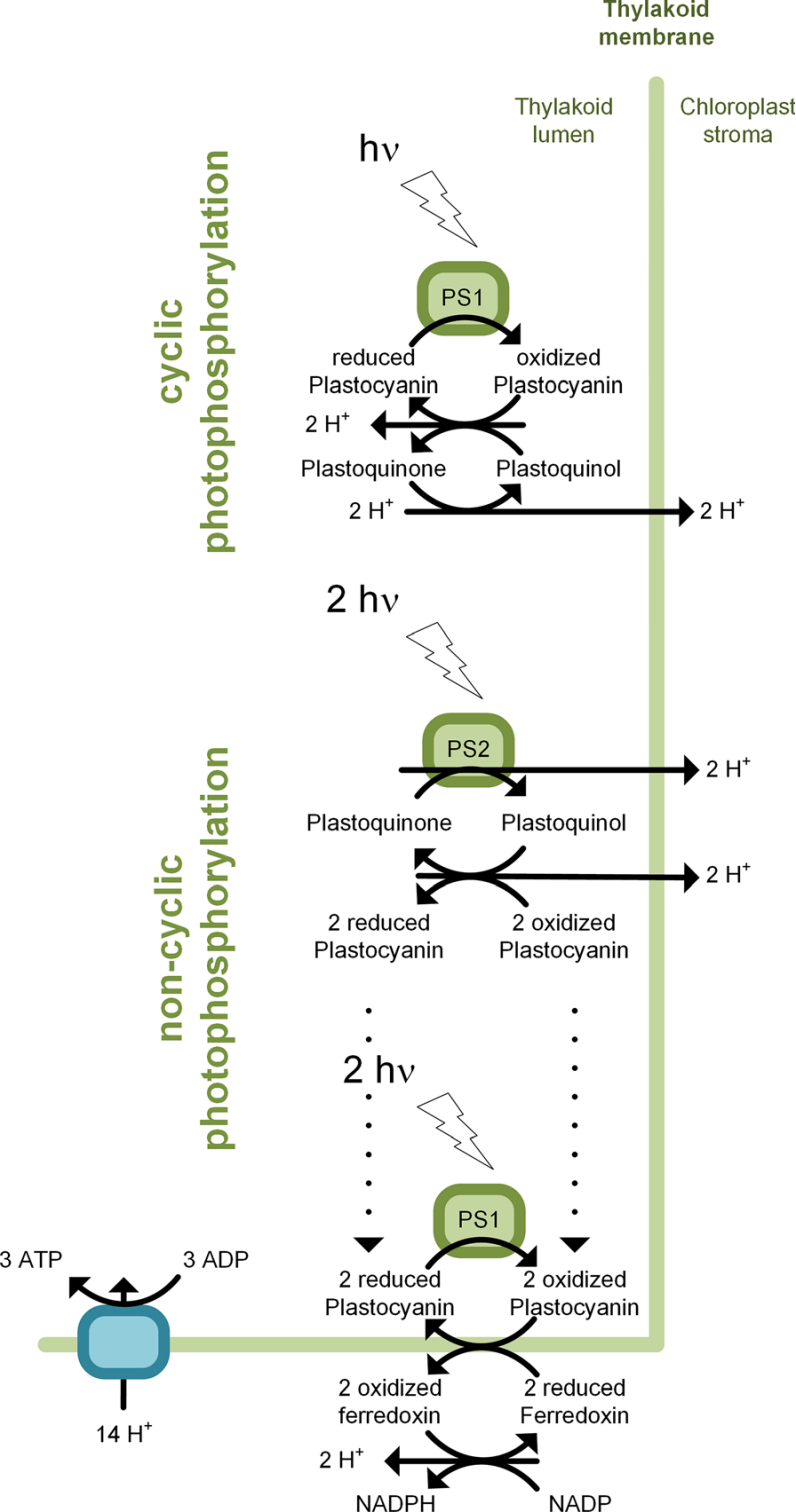
Cyclic and non-cyclic photophosphorylation in plants. A standard stoichiometric ratio between cyclic and non-cyclic photophosphorylation of 2:1 was assumed for most simulations. It accounts for 2x4 photons for non-cyclic electron flow and for one absorbed photon for cyclic photophosphorylation, respectively. In addition, simulations with a varied ratio were conducted in order to study the existing plasticity of cyclic and non-cyclic photophosphorylation (see Fig. 8)

The created metabolic network of *A. thaliana*’s core carbon pathways reflected 511 metabolic conversions in total (Fig. 1). In total, 511 metabolic reactions are accounted for: 82 cytosolic, 348 plastidic, 27 mitochondrial and 6 peroxisomal reactions (see Additional file 2). Additionally, 41 reactions describe intracellular transport and 5 reactions mediate the uptake of nutrients. The derived network is descriptive for about 1567 metabolic genes, all known to exist in *A. thaliana* (http://pmn.plantcyc.org). The chosen topology is specific for green leaf tissue. The network contained the Calvin-Benson-Bassham (CBB) cycle for CO_2_ assimilation and photosynthesis, the gluconeogenesis, the glycolytic Embden-Meyerhof-Parnas (EMP) pathway, the oxidative and the non-oxidative pentose phosphate (PP) pathway, the tricarboxylic acid (TCA) cycle, the reactions of photorespiration, of starch biosynthesis and degradation, of energy metabolism, as well as the anabolic pathways for biomass synthesis (Figs. 1 and 2). The latter considered compartment-specific supply of the individual precursors (Additional file 1: Table S1 and S2) [17, 30]. Internal starch, degraded in green tissue in the dark, and atmospheric CO_2_, assimilated in the light, were included as natural carbon sources. The network model specifically included metabolic conversions that contribute to leaf growth, whereas growth-unrelated metabolic processes, e.g. chemical defense, were omitted. For the simulations, the model was condensed. Single reactions in linear pathways and stoichiometrically identical parallel pathways were lumped together. The condensation was crucial to enable the computationally demanding simulation of the entire set of elementary flux modes. In this way, the total equation system to describe the plant metabolism could be represented in a compact form by only 129 reactions and 70 metabolites (Fig. 1, Additional file 1: Table S3 and S4, Additional file 3). The condensed model remained fully descriptive of the entire set of metabolic conversions, as demonstrated by additional simulations (Additional file 1: information S1). Among the reactions, 34 reactions belonged to inter-compartmental and to extracellular transport. Based on the information provided in the above-mentioned databases and in the literature, 63 reactions were constrained as irreversible (Additional file 1: Table S5). The condensed model topology allowed the computationally demanding calculation of all feasible metabolic routes in the leaf, through enumeration of elementary flux modes. The model was validated and fine structured with regard to the presence, location and thermodynamic properties of individual reactions, based on experimental evidence, as described below.

### Compartmentation

The network reflected the four major compartments of plant leaves that contribute to biochemical conversions: cytosol, peroxisome, mitochondrion and plastid (http://metacrop.ipk.gatersleben.de) (http://pmn.plantcyc.org) [31, 32, 45–48]. The cytosol comprised the reactions of the EMP pathway [46], the oxidative part of the PP pathway [45] and the reactions of starch degradation from maltose [31]. The plastid contained the photosynthetic CBB cycle, a second copy of the EMP pathway [46], the oxidative and the non-oxidative PP pathway [45] and the starch metabolism [31]. The TCA cycle was assigned to the mitochondrion [47]. The photo-respiratory system, known to be a multi-compartment process, was distributed accordingly across plastid, peroxisome and mitochondrion [49]. Additionally, each compartment contained malate dehydrogenase (http://metacrop.ipk.gatersleben.de) [50]. Pyruvate kinase was considered as cytosolic and as plastidic reaction [46], whereas the pyruvate dehydrogenase complex was assigned to the mitochondrion and to the plastid [47]. The supply of cytosolic acetyl-CoA was attributed to ATP-citrate lyase in the cytoplasm, which uses citrate as a substrate [51]. Malic enzyme, specific for photosynthetic tissue, was integrated into the plastid [46].

### Inter-compartmental and external transport

The separation of the metabolic routes in distinct organelles requires the translocation of specific compounds across cellular membranes. Based on experimental evidence, unidirectional or bidirectional transport between cytosol and mitochondrion was assumed for pyruvate, malate, inorganic phosphate, glycine and serine, respectively, whereas antiporters were considered for malate/oxaloacetate, citrate/oxaloacetate and ATP/ADP [34, 52–55]. Transport reactions between the cytosol and the plastid were implemented for 3-phosphoglycerate, glycerate, glycolate, malate/oxaloacetate, pyruvate, phosphoenolpyruvate, xylulose 5-phosphate, glucose 6-phosphate, dihydroxyacetone phosphate, maltose, ATP/ADP and inorganic phosphate [34, 45, 56–59]. Hereby, the translocation of phosphorylated carbohydrates across the plastid membranes was linked to the simultaneous antiport of inorganic phosphate [57]. In addition, active peroxisomal membrane transfer of malate/oxaloacetate, glycerate, glycolate, glycine and serine was considered [49, 50]. So far, a transporter for acetyl-CoA has not been discovered and was therefore not incorporated [60]. CO_2_ was assumed to freely diffuse within the cell [61], photons were capable of penetrating both the extracellular and plastidic membranes [62] and inorganic phosphate was available from the vacuole [63].

### Energy Household

Redox, energy and phosphate metabolism were compartmentalized across the different organelles. This included the confinement of the photosynthetic light reactions to the plastid and of the oxidative phosphorylation system to the mitochondrion. A vacuolar storage pool for inorganic phosphate was considered [63]. Inorganic phosphate could be transported by specific carriers between the cytosol and both the plastid and the mitochondrion. ATP/ADP antiporters were incorporated into the mitochondrial and the plastidic membrane, respectively. In addition, the exchange of reducing equivalents between cytosol and plastid, mitochondrion and peroxisome was attributed to malate dehydrogenase, which was coupled to malate and oxaloacetate channeling across the organelle barriers. To account for widely abundant isoenzymes, capable of utilizing either NADPH or NADH or both molecules as cofactor, an oxidoreductase for interconversion of NADPH and NADH was included in the cytosol [64]. This compartmented consideration is more realistic. Additional simulations of a simplified network without this strongly compartment-specific energy, redox and phosphate metabolism showed that the maximum biomass formation was not affected by the compartmentalized energy, redox and phosphate acquisition (data not shown). This demonstrates that the network allows for full equilibration of energy, redox and phosphate among the compartments. The following considerations were additionally included to implement energy efficiency. The ratio of ATP: NADPH, produced in a photosynthetic cell, depends on the generally accepted plasticity of the photosynthetic light reactions for energy production (Fig. 2) [65–69]. As it is still unresolved, how this mechanism functions exactly, an average ATP to NADPH ratio of 1.5 was usually chosen, which accounts for cyclic electron flow around photosystem I of two photons and of non-cyclic electron flow caused by eight photons [65]. In a set of additional simulations, the photosynthetic plasticity was investigated by varying the ratio between cyclic and non-cyclic electron flow, i.e. the ATP to NADPH ratio (see below). In order to provide sufficient ATP for maintenance, a surplus of ATP was set as constraint for the modelling [70]. This excluded unrealistic scenarios, which would have produced less ATP than needed for growth, but allowed for scenarios, which produced an apparent excess of ATP to serve for maintenance purposes.

### Anabolic pathways

The biochemical composition of *A. thaliana* leaves was collected from previous work (Additional file 1: Table S1). Nearly half of cellular carbon is stored in the cell wall [71–73], whereas one third is contained in proteins [74]. Based on experimental data, the remaining carbon was distributed among lipids [75–77], carbohydrates [71, 74, 78], porphyrins [79, 80] and other biomass components [74, 81–83]. The anabolic pathways form the most important carbon sink during growth. As most of these peripheral biosynthetic pathways, are linear and a metabolic steady-state is assumed, they can easily be summarized into a single, lumped biomass equation, starting from 12 central metabolic precursor metabolites (Additional file 1: Table S2), without noteworthy degeneration of information content (Additional file 1: Information S1). The intracellular localization of the individual biosynthetic enzymes determined from which organelle a particular precursor originated. For instance, aromatic amino acids are synthesized from phosphoenolpyruvate and erythrose 4-phosphate in the plastid [84], whereas cellulose originates from hexose 6-phosphate in the cytosol [85].

### Plant metabolism involves highly efficient carbon assimilation and conversion

Plants are subjected to changing environmental conditions, most prominently the light–dark shift. During the day, light is available as copious source of energy, allowing photosynthetic carbon assimilation, whereas during the night, the breakdown of internal starch delivers the necessary energetic power [86]. From a metabolic engineering perspective, knowledge on both physiological states is essential to optimize plants in a way that carbon is channeled optimally towards desired traits throughout the diurnal cycle. Therefore, these two fundamental growth states, light and dark metabolism, were now studied using elementary flux mode analysis.

The full sets of elementary flux modes, which span the entire space of feasible flux distributions, were calculated both for metabolism in the light using CO_2_ as sole carbon source (light-metabolism), and for metabolism in the dark using internal starch (dark-metabolism). The solution space consisted of 1.2 million elementary flux modes for the light-metabolism and of 5.7 million elementary flux modes for the dark-metabolism (Table 1). The theoretical maximum growth yields were 27.4 and 28.6 (g biomass) (C-mol substrate)^−1^ for the two scenarios. Assuming equal contribution of day and night metabolism, the resulting mean value of 28.1 (g biomass) (C-mol substrate)^−1^ closely resembles experimental values for *A. thaliana* leaves of about 25.2 (g biomass) (C-mol substrate)^−1^ [87]. The maximum theoretical biomass formation, predicted by the model, revealed a high carbon efficiency of plant metabolism: 86.8 to 90.6 % of the assimilated carbon was incorporated into biomass (Table 2). For prolonged illumination phases, the simulated values further approached the measured ones (Additional file 1: Table S6). This was a first indication that in vivo plant metabolism closely approaches theoretical optimum performance with regard to stoichiometric capacity, which seems a particularly useful characteristic with regard to use in production. When the light influx was omitted from the model, no modes resulted for CO_2_ assimilation, deducing that degradation of a heterotrophic substrate, such as starch, is the only feasible phenotype in the dark.

**Table 1:** Outline of fundamental physiological states in plant leaves and their accompanying elementary flux modes (EFMs)

	Autotrophy		Heterotrophy	
	Light	Dark	Light	Dark
CO_2_ Uptake	+	+	-	-
Starch Degradation	-	-	+	+
Light influx	+	-	+	-
Number of EFMs	1 206 894	11	11 296 607	5 653 544

**Table 2:** Physiological parameters of *Arabidopsis* leaves

	light-metabolism model	dark-metabolism model	Whole plant experiment
Substrate	CO_2_	C12-starch subunit	CO_2_
Substrate Uptake [mmol substrate]	1	1	1
Biomass Production [mg Biomass]	28.6	329	25.2
Biomass Yield [g/C-mol Substrate]	28.6	27.4	25.2
Carbon Efficiency [%]	90.6	86.8	79.9

Maximal theoretical biomass production of Arabidopsis leaves as predicted by the metabolic model, both in the light and in the dark. The simulation data are compared with the experimentally determined growth yield of Arabidopsis rosettes [87]. Details on the experimental yield calculation can be found in Additional file 1: Table S6

### A narrow range of absorbed photons supports optimal plant growth

**Fig. 3:**
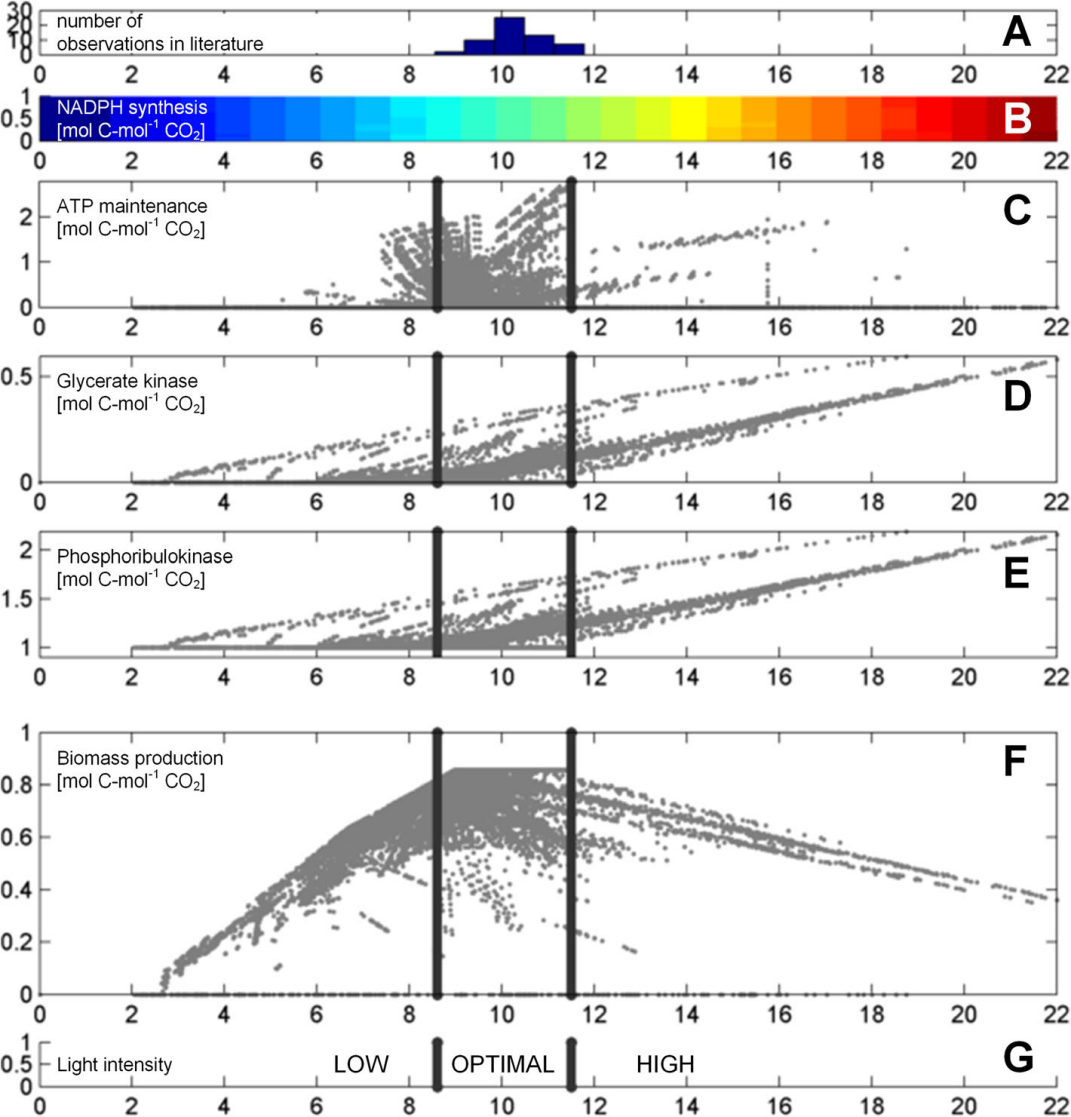
Light quantum requirement for plant growth. Histogram of the quantum yield values for C_3_ plants under ambient atmospheric conditions (**a**). The values were taken from previous work [43], which reviewed the in vivo range of the quantum yield in C_3_ and C_4_ plant lineages. Not all harvested quanta are converted into chemical energy as some are lost through absorption by pigments, which are unable to contribute their excitation energy to photosynthesis. Therefore, the nominated range was corrected assuming 47 % of photons are outside the photosynthetically active range [44]. The linear relationship between modelled NADPH synthesis and quantum requirement is visualized as a color scale between blue (low) and red (high) (**b**). Modeled ATP maintenance production associated with the observed light influx (**c**). Modeled glycerate kinase activity (**d**) and phosphopentokinase activity (**e**) as a function of photon influx. Observed biomass formation in relation to the quantum requirement (**f**). Subdivision of the data into three light regimes (**g**). Energetically inefficient modes that included massive cycling of resources were eliminated from the analysis. Although stoichiometrically possible, these modes represent futile cycling of carbon across the internal membranes to dissipate energy, which was considered energetically inefficient

The computed set of elementary modes was used to investigate the impact of the light influx on growth in more detail. First, energetically inefficient modes that included massive cycling of resources were eliminated. Although stoichiometrically possible, these modes involve futile cycling of carbon across the internal membranes to dissipate energy (Additional file 1: Figure S1). They are an emergent property, which arises from the compartmentation of metabolic pathways into intracellular organelles. Because such massive futile cycling is energetically inefficient, they are considered both evolutionary and physiologically implausible [88, 89]. Furthermore, the high reaction rates, observed in such elementary modes, would require the additional synthesis of large amounts of protein with further energetic burden, further justifying the elimination of these modes from the subsequent evaluation. This identified a physiologically plausible subset of 848,629 individual flux modes. When these modes were mapped against the corresponding acquired light influx for each of the modes (Fig. 3f), the production of biomass was found highest in a defined range between 8.6 and 11.5 (mol photons) (mol CO_2_)^−1^. This corresponds to a quantum yield between 0.09 - 0.12 (mol CO_2_) (mol photons)^−1^, obviously supporting optimal photosynthesis.

### Flux rearrangement between optimum day and night metabolism requires reversible translocation of carbohydrates

**Fig. 4:**
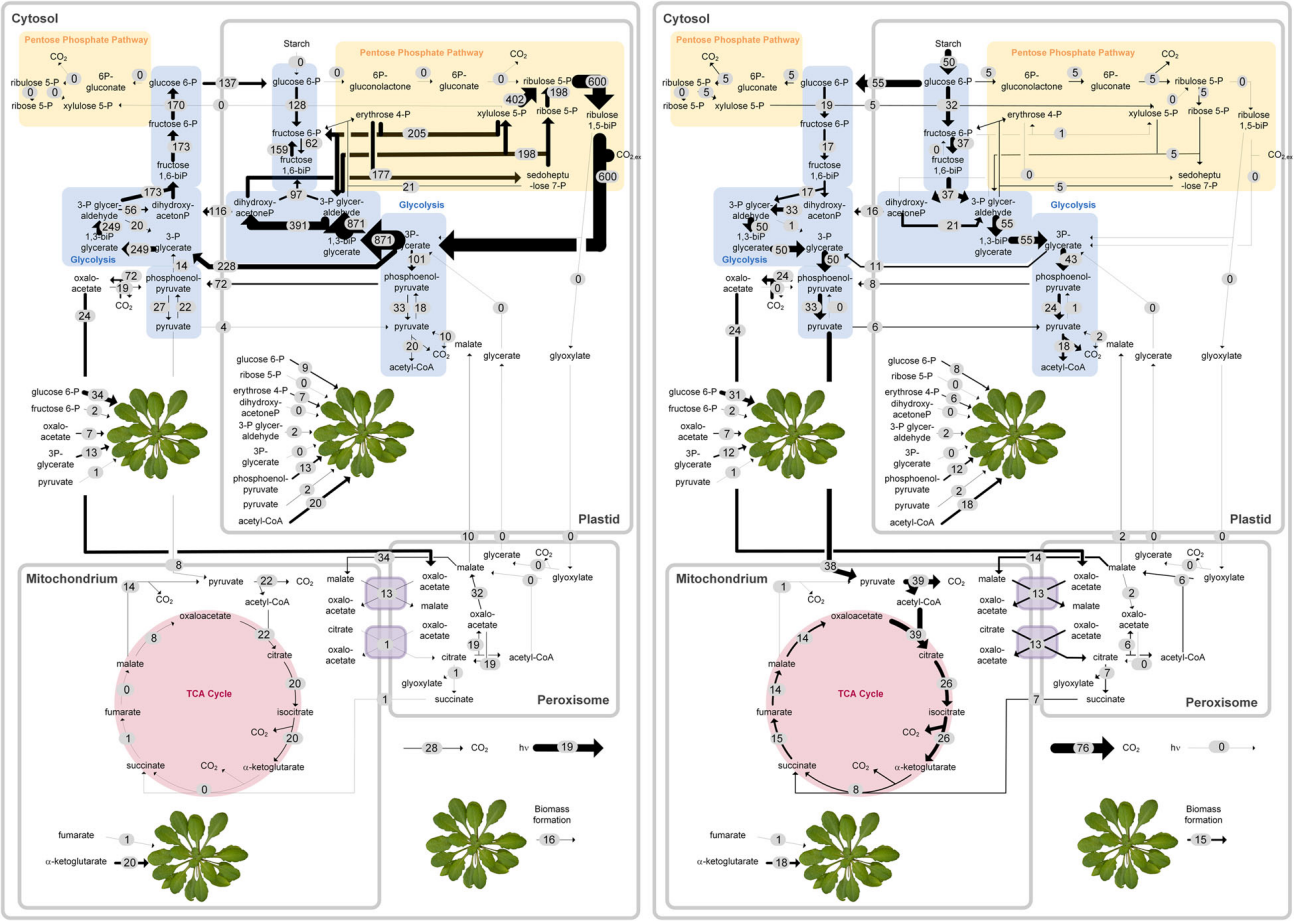
Relative intracellular fluxes of day and night metabolism in *A. thaliana* leaves as predicted from elementary flux mode simulation. The value on the arrow and the thickness of the arrow represent the average flux value of the top 1 % biomass producing modes of *Arabidopsis* leaves in the light (left) and in the dark (right), as predicted by the simulation, excluding energetically inefficient modes. All fluxes are normalized to the substrate uptake flux and are given in mol mol^−1^ substrate. To enable a direct comparison, flux values, linked to light-metabolism are normalized to 100 mol of CO_2_ uptake and flux values, linked to dark-metabolism are normalized to 50 mol of starch degradation, as starch is represented by carbon twelve dimers. The arrow thicknesses are given in log-scale. The arrows pointing towards the *Arabidopsis* rosettes visualize the amount of building blocks for biomass synthesis. Biomass (BM) synthesis in expressed in g BM

**Fig. 5:**
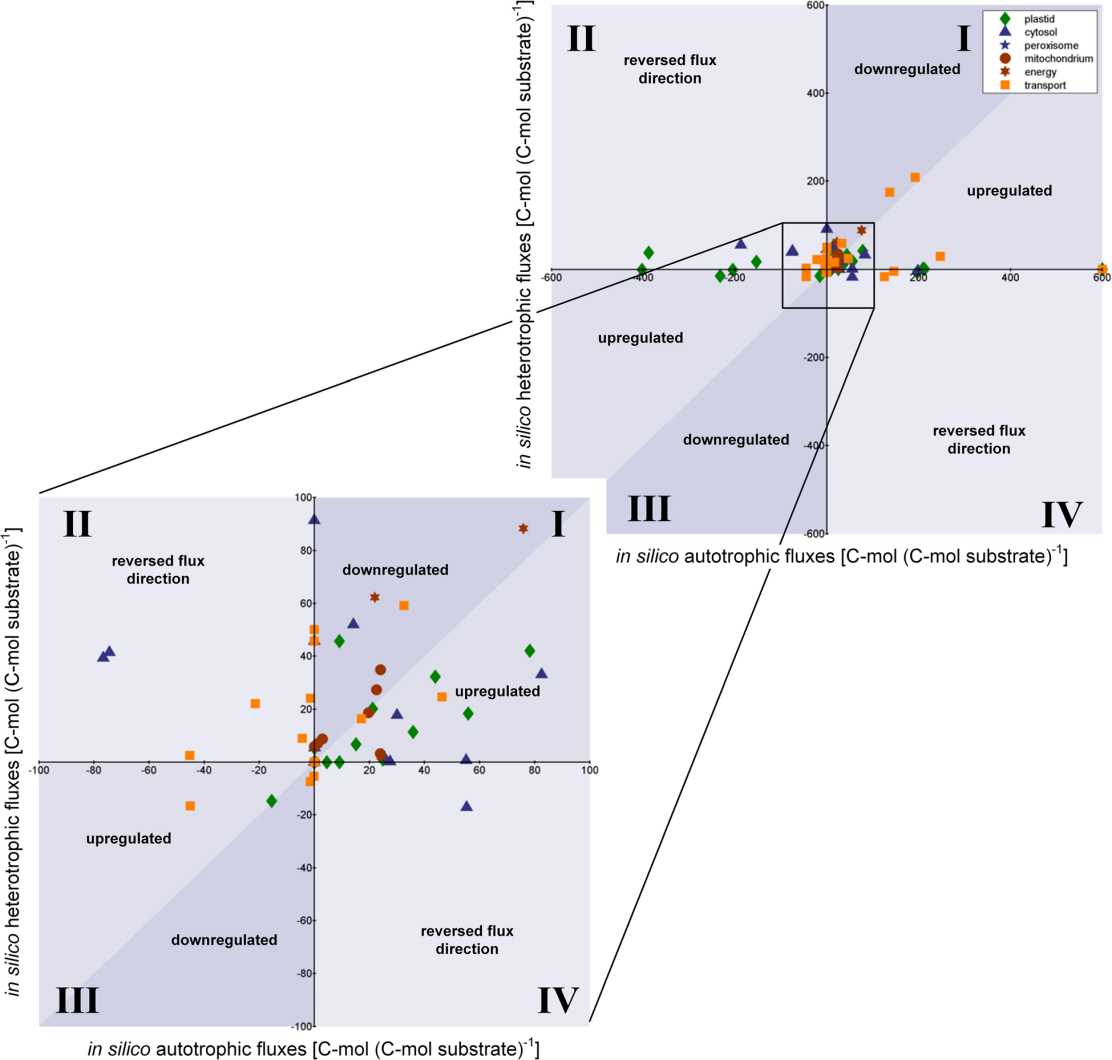
Autotrophic versus heterotrophic fluxes. Plant metabolic flux changes linked to the switch from optimal light-metabolism to optimal dark-metabolism. The given flux values refer to the average among the top 1 % biomass producing modes. The second and the fourth quadrant, respectively, depict reactions, which respond to the switch by a reversed flux direction. The first and third quadrant, respectively, are subdivided into reactions that are up-regulated or down-regulated during the light phase. All fluxes are expressed in C-mol C-mol^−1^

Next, day-time and night-time metabolism were evaluated on the level of intracellular fluxes. The flux distributions, which resemble optimum growth, revealed large differences between the two conditions (Figs. 4 and 5). Shortly, dark metabolism involved the degradation of starch into maltose, which was subsequently transported across the plastid membrane (Fig. 4). In the cytosol, maltose was hydrolyzed into two glucose molecules, followed by phosphorylation to glucose 6-phosphate. The sugar phosphate was then channeled into the cytosolic and the plastidic EMP pathways. The glucose 6-phosphate translocator mediated the distribution of carbon between the different compartments. In this way, both malate and pyruvate were generated through malate dehydrogenase and through the EMP pathways, respectively, and were subsequently imported into the mitochondrion to fuel the TCA cycle. The light-metabolism assimilated atmospheric CO_2_ via the CBB cycle in the plastid (Fig. 4). This involved a high flux through the CBB cycle and the non-oxidative PP pathway, as both routes are strongly entwined. Mainly, the triose-phosphate translocators supplied carbon-building blocks into the cytosol, whereby the glucose 6-phosphate translocator recycled carbon back into the plastid. Mostly, malate was built from initial carbon conversions and it was then imported into the mitochondrion. Malic enzyme provided the TCA cycle with pyruvate. In this regard, the translocation of pyruvate between the different compartments was one prominent example of a fully reversed flux, as described above. To conclude, in the light, especially the plastidic metabolism was highly active and the triose phosphate carriers transported carbon out of the plastid. Mainly malate was imported into the mitochondrion, whereas in the dark both malate and pyruvate were channeled across the mitochondrial membrane and the cytosol showed slightly more activity than the plastid. The main carbon export from the plastid occurred in the form of maltose. The most significant differences between light and dark metabolism were found for the plastid metabolism, for the energy metabolism and for specific inter-compartmental transporters (Fig. 5). In contrast, especially the mitochondrial and the peroxisomal reactions remained rather unchanged. The simulation data predict an on/off shift for five previously postulated diurnal switches, i.e. ribulose 1,5-bisphosphate carboxylase, phosphoribulokinase, fructose 1,6-bisphosphate phosphatase, NADP-dependent glyceraldehyde 3-phosphate dehydrogenase and sedoheptulose 1,7-bisphosphate phosphatase [90]. In addition, many more changes were observed. A reversal in flux direction resulted for no less than 19 reactions (Additional file 1: Table S7, Fig. 5), whereas 70 reactions kept the direction of flux, but showed significant changes in flux value. From the latter, 66 fluxes were significantly decreased for the dark metabolism as compared to the light metabolism, whereas 23 reactions showed a significant increase (Fig. 5). Given the fact, that the analysis was on the averaged flux behavior of the top 1 % biomass producing modes, it cannot be excluded that alternative solutions exist within the flux solution space that do not behave in the same way as the here described flux changes (Additional file 1: Figure S3).

### Simulation quantifies differences in energy and redox supply between light and dark metabolism

**Fig. 6:**
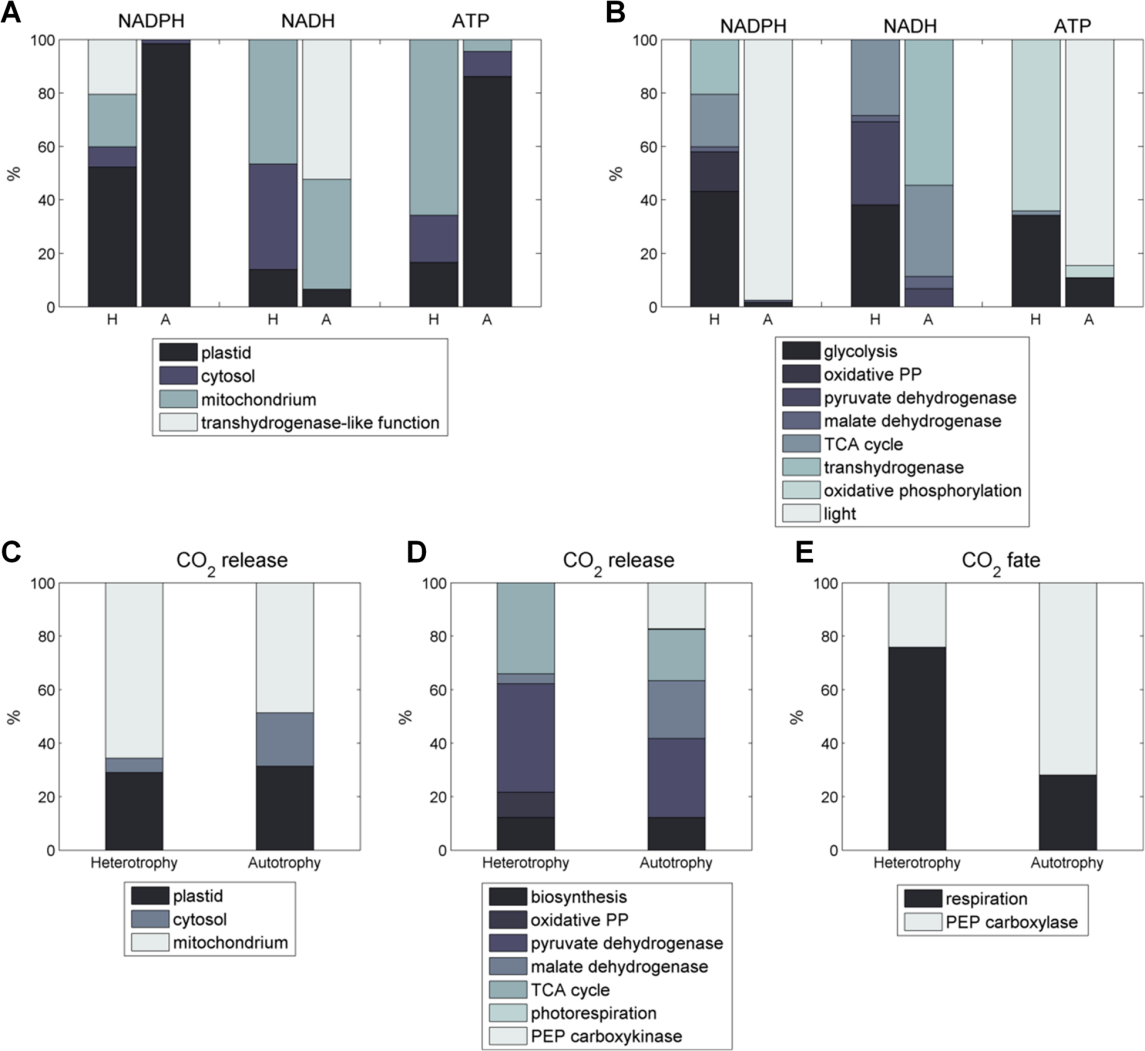
Metabolic properties of light and dark metabolism. Metabolic properties of the intracellular flux distributions in the light and in the dark under optimum conditions, respectively, as calculated by elementary flux analysis (average flux values from the top 1 % biomass producing modes, predicted by the EFM simulation. **a**-**b** Redox and energy production expressed per total redox and ATP-demand. **a** Pathway-based subdivision and (**b**) compartment-based visualization. **c**-**e** Details about origin and fate of CO_2_ as fraction of the total CO_2_ released. Here, the fluxes were subdivided into different (**c**) compartments and (**d**) pathways

As shown in the previous section, the metabolism in the dark recruited all compartments to supply ATP and reducing power, whereas the plastid was the dominant compartment in the light (Fig. 4). Next, we analyzed in more detail, which metabolic processes are responsible for the supply of energy and reducing power. The analysis again considered the optimal dark and light fluxes, i.e. the average of the top 1 % biomass producing modes. Illuminated leaves largely delivered NADPH and ATP through the photosynthetic light reactions in the plastid (Fig. 6), whereas the NADH stemmed mostly from cytosolic malate dehydrogenase, i.e. was exported as NADH from the plastid [64]. During the dark, ATP was produced predominantly via oxidative phosphorylation in the mitochondrion (60 %), and reduced NADPH was generated by all compartments. Secondly, the formation and assimilation of carbon dioxide was different between the two physiological states (Fig. 6). Generally, the total net flux of CO_2_ production was higher for the dark-metabolism (10 %), as compared to light-metabolism (5 %), whereby overall, both values were rather low. It was interesting to note that also the origin of the released CO_2_ differed. Under both conditions, the carbon loss by photorespiration was negligible, whereas the largest contributors to CO_2_ release were the TCA cycle and pyruvate and malate dehydrogenase. During the dark, additional carbon was released during the generation of NADPH in the oxidative PP pathway, whereas during illumination, previously assimilated CO_2_ was lost via PEP carboxykinase.

### Optimum anabolic pathway use switches between day and night

**Fig. 7:**
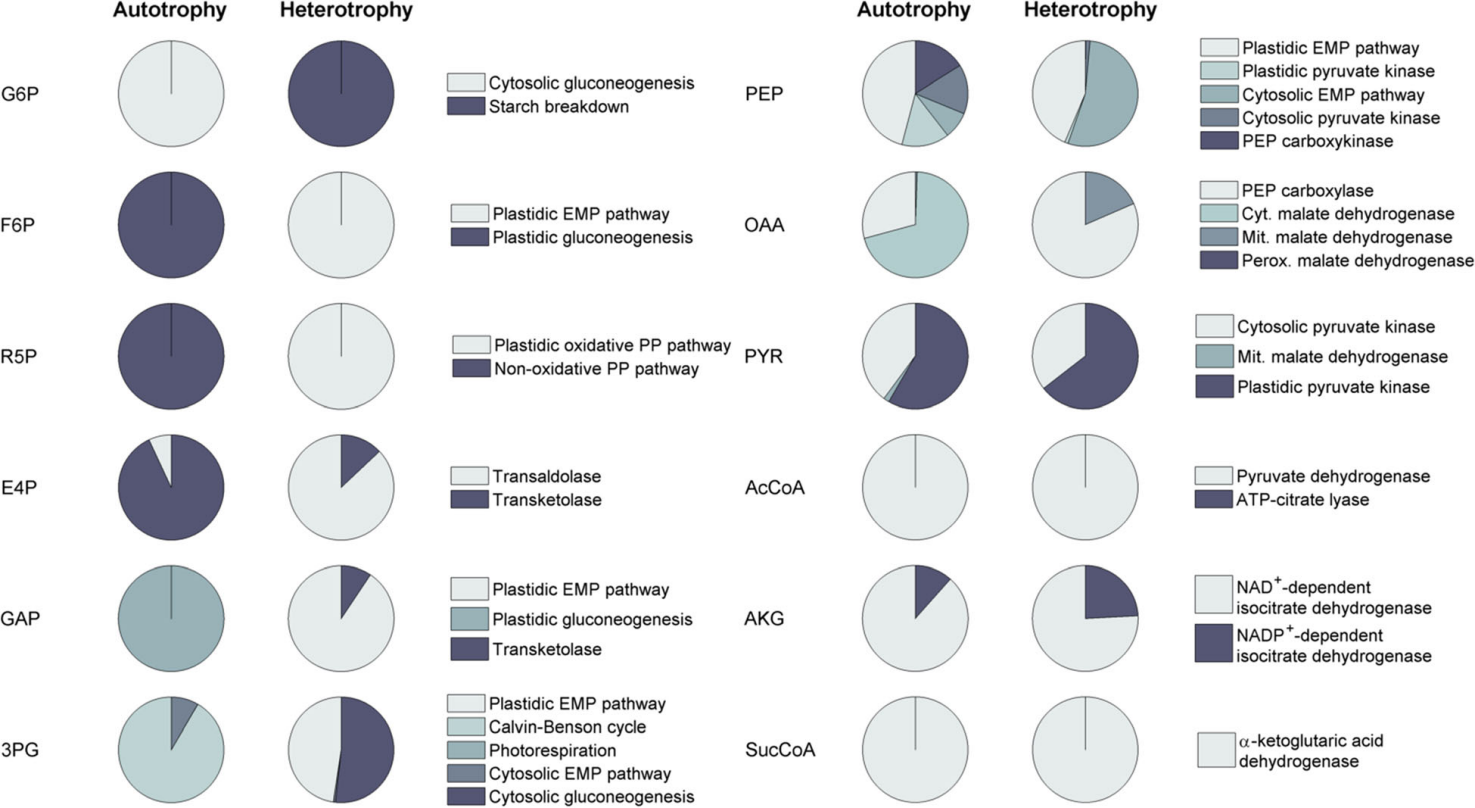
Pathways involved in the allocation of anabolic precursors. Relative contribution of individual pathways to supply anabolic precursors in the light and in the dark under optimum conditions (average flux values from the top 1 % biomass producing modes, predicted by the EFM simulation). Abbreviations: 3-phosphoglyceric acid (3PG), acetyl-CoA (AcCoA), α-ketoglutaric acid (AKG), erythrose 4-phosphate (E4P), fructose 6-phosphate (F6P), glucose 6-phosphate (G6P), glyceraldehyde 3-phosphate (GAP), oxaloacetate (OAA), phosphoenolpyruvate (PEP), pyruvate (PYR), ribose 5-phosphate (R5P), succinyl-CoA (SucCoA)

The striking differences in the localization of energy and reducing power supply among the cellular compartments now suggested to also inspecting anabolic metabolism. Therefore, the metabolic pathways, responsible for the synthesis of each anabolic precursor, were investigated and compared between the optimum growth modes in the light and in the dark (average flux values of the top 1 % biomass producing modes). Interestingly, the biosynthetic origin of many of the twelve anabolic precursors strongly depended on the physiological growth mode, i.e. light and carbon acquisition (Fig. 7). As example, the majority of 3-phosphoglycerate in the light was supplied via the CBB cycle, whereas, in the dark, the precursor exclusively stemmed from the EMP pathway. In addition, ribose 5-phosphate was produced through the non-oxidative PP pathway under illumination, whereas, in the dark, it was mainly generated by the oxidative PP pathway. For other precursors, such as α-ketoglutaric acid, the metabolic network recruited isoenzymes, which differed in cofactor use, depending on the illumination conditions.

### Pathway fluxes remain stable upon photosynthetic plasticity

**Fig. 8:**
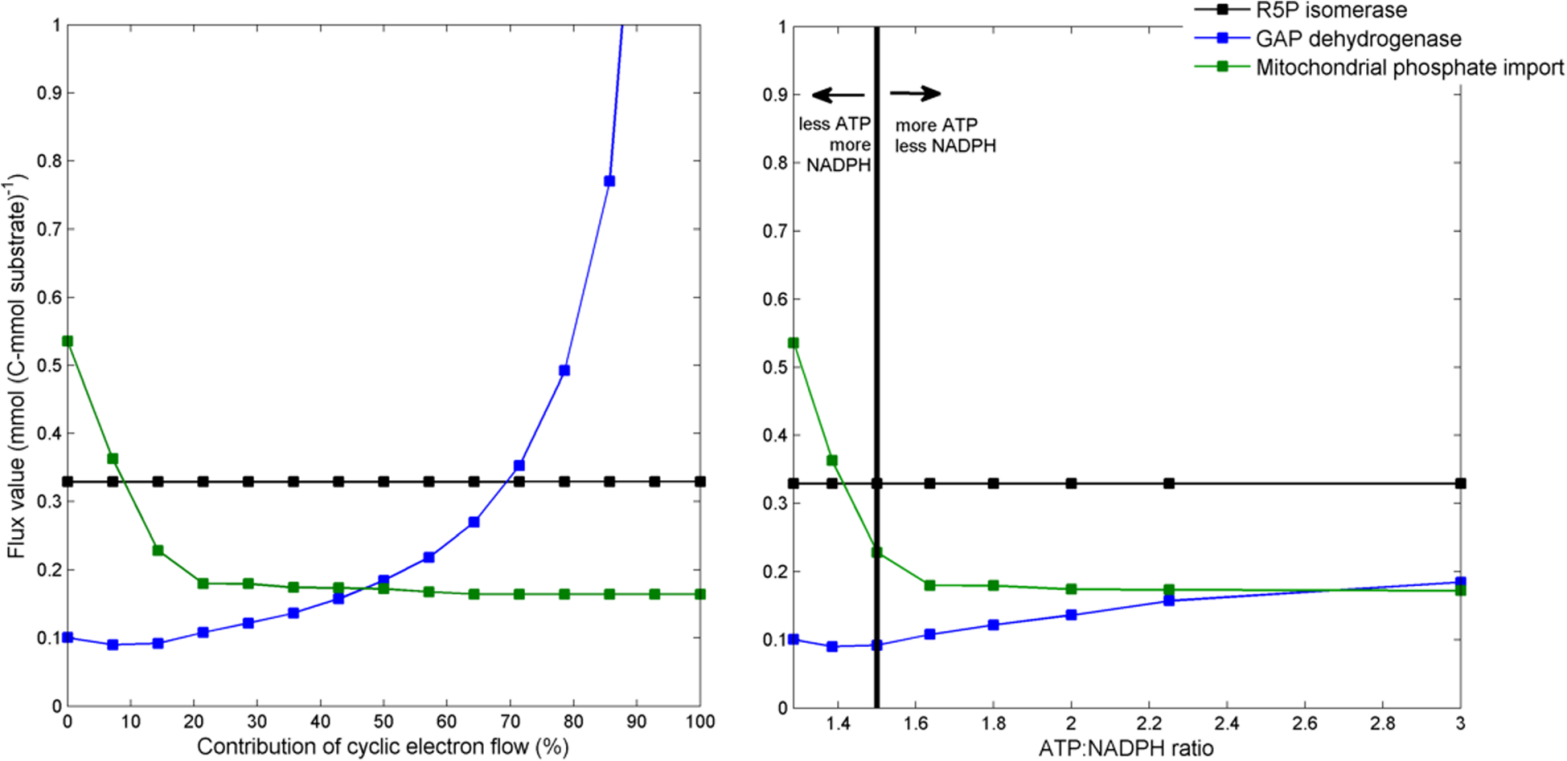
Impact of the cyclic electron flow to photophosphorylation on intracellular metabolic fluxes in *A. thaliana*. Trends in flux changes of optimal fluxes as calculated for increasing contribution of cyclic electron flow to photophosphorylation (left) and increasing ATP:NADPH ratio (right). How electron flow and ATP: NADPH ratio relate, is described in Additional file 1: Table S8. Optimal growth was considered by averaging the flux value of the top 1 % biomass producing modes, predicted by the simulation of the elementary modes

It is generally accepted that cyclic and non-cyclic electron flow both contribute to the in vivo photosynthetic light reaction [65–67]. However, it is unclear at which ratio cyclic and non-cyclic electron flow co-operate. It is hypothesized that the observed flexibility might allow the modulation of the ATP: NADPH ratio to match the demand of metabolism under changing environmental conditions [66, 67], as non-cyclic electron flow allows for simultaneous NADPH and ATP synthesis, whereas cyclic electron flow solely generates ATP. It is prevalent in the literature that under environmental stress, such as cold, light or osmotic stress, certain metabolic changes arise, that modulate the cellular ATP: NADPH demand [91, 92]. In this regard, it has been acknowledged, that this modulation requires fine-tuned regulation of the contribution of cyclic electron flow to photosynthesis [93]. Reversely, sudden light fluctuations, such as shading by a cloud or overhanging leaf during full sunlight at noon, directly cause changes in the contribution of cyclic electron flow to photosynthesis [94]. In particular, an increase in cyclic electron flow appears important in protection of photosystem I against photo inhibition [94, 95]. It was now interesting to investigate the interplay between the metabolic pathways in meeting different ratios for NADPH and ATP, as expected under sudden fluctuations in light. For this purpose, optimal flux distributions (In average flux values of the top 1 % biomass producing modes) of light-metabolism were calculated for 15 scenarios with increasing contribution of cyclic electron flow to the overall electron flow, i.e. an increasing ATP:NADPH ratio. Ratios between non-cyclic and cyclic electron flow of 14:0, 13:1, 12:2, 11:3, 10:4, 9:5, 8:6, 7:7, 6:8, 5:9, 4:10, 3:11, 2:12, 1:13 and 0:14 were considered (Additional file 1: Table S8). For all ratios, except for 100 % cyclic electron flow, the same theoretical maximum for biomass formation was found. Omission of non-cyclic electron flow did not allow for any biomass formation. Three general trends could be observed when plotting the absolute flux values for optimal growth against the ratio between cyclic and non-cyclic electron flow (Fig. 8). Either, (i) optimal flux values were not influenced by the type of phosphorylation (e.g. ribose 5-phosphate isomerase) or an exponential increase at either (ii) extremely low or (iii) extremely high contribution of cyclic electron flow was observed (e.g. mitochondrial phosphate import and glyceraldehyde 3-phosphate dehydrogenase, respectively). Between 20 and 70 % cyclic electron flow contribution all fluxes remained rather unchanged, which can be taken as an indication for a highly flexible plant metabolism, easily capable of adjusting to different ATP:NADPH ratios. The specific metabolic flux changes that allow to cope with an increase in cyclic electron flow, constitute futile cycling of metabolites to actively dissipate an excess in ATP (Additional file 1: Figure S2). Examples include, substrate cycling between phosphoenolpyruvate and pyruvate, substrate cycling between fructose 1,6-bisphosphate and fructose 6-phosphate, cycling across the plastid membrane mediated by 3-phosphoglycerate and glucose 6-phospate transport and the activity of the cytosolic NADPH-dependent glyceraldehyde 3-phosphate dehydrogenase. Furthermore, the metabolic fluxes that are most influenced by a decreased ATP: NADPH ratio, appear to handle an excess of redox power by channeling NADPH through the malate/oxaloacetate shuttle into the mitochondrion, where ATP is produced by oxidative phosphorylation (Additional file 1: Figure S2). This supports the hypothesis that mitochondria can act as a sink for reduced NADPH [96–98]. In addition, plastidic triose phosphate is increasingly exported to the cytosolic EMP pathway, thus effectively reducing the plastidic ATP requirement.

## Discussion

### Systematic analysis of the elementary mode solution space unravels the light stress response

The constructed metabolic network enabled the adequate description of fundamental physiological traits, previously found experimentally for *A. thaliana,* such as growth and photosynthetic efficiency, which can be taken as indication of its high quality and validity (Fig. 3 and Table 2). Obviously, *Arabidopsis* rosettes operate close to optimum. This becomes evident from the resemblance between the predicted theoretical maximum and the experimental growth yield (Table 2) and the predicted optimum range of photon absorbance, which rather exactly match with the range observed in vivo (Fig. 3) [39–43].

Optimal growth only results for a narrow range of photon absorbance, while higher and lower light intensities reduce growth efficiency, respectively (Fig. 3). Possible molecular reasons for this phenomenon can be directly extracted from the flux mode solution space. The direct product of photo-reduction is NADPH (Fig. 3b). Under high light intensity, its production soon exceeds the biosynthetic need. A physiologically feasible way to cope with such NADPH excess is an increased flux through the photo-respiratory pathway, as photorespiration consumes NADPH. Interestingly, a clear relationship between the flux through the photo respiratory system (glycerate kinase) and phosphoribulokinase, and the quantum requirement under high light intensity suggests that indeed *Arabidopsis* deals with light stress by increasing photorespiration (Fig. 3d-e). A direct consequence of such an up-regulation is reduced growth, because photorespiration is associated with a net loss of carbon as CO_2_ [99–101]. Recently, an activated photorespiration has been experimentally recognized in plants as an important light stress response to dissipate excess reducing equivalents and energy [102]. Alternatively, NADPH excess could also be handled by an increased consumption of ATP, e.g. through futile cycles. From a metabolic viewpoint, this would involve a conversion of excess NADPH into NADH by a transhydrogenase-like reaction, and fueling the phosphorylation of ADP into ATP. In this way, growth could be maintained at its optimal rate, however in an energetically less efficient way.

### The intracellular pathways of Arabidopsis rosettes operate in vivo near the predicted optimum flux distribution

**Fig. 9:**
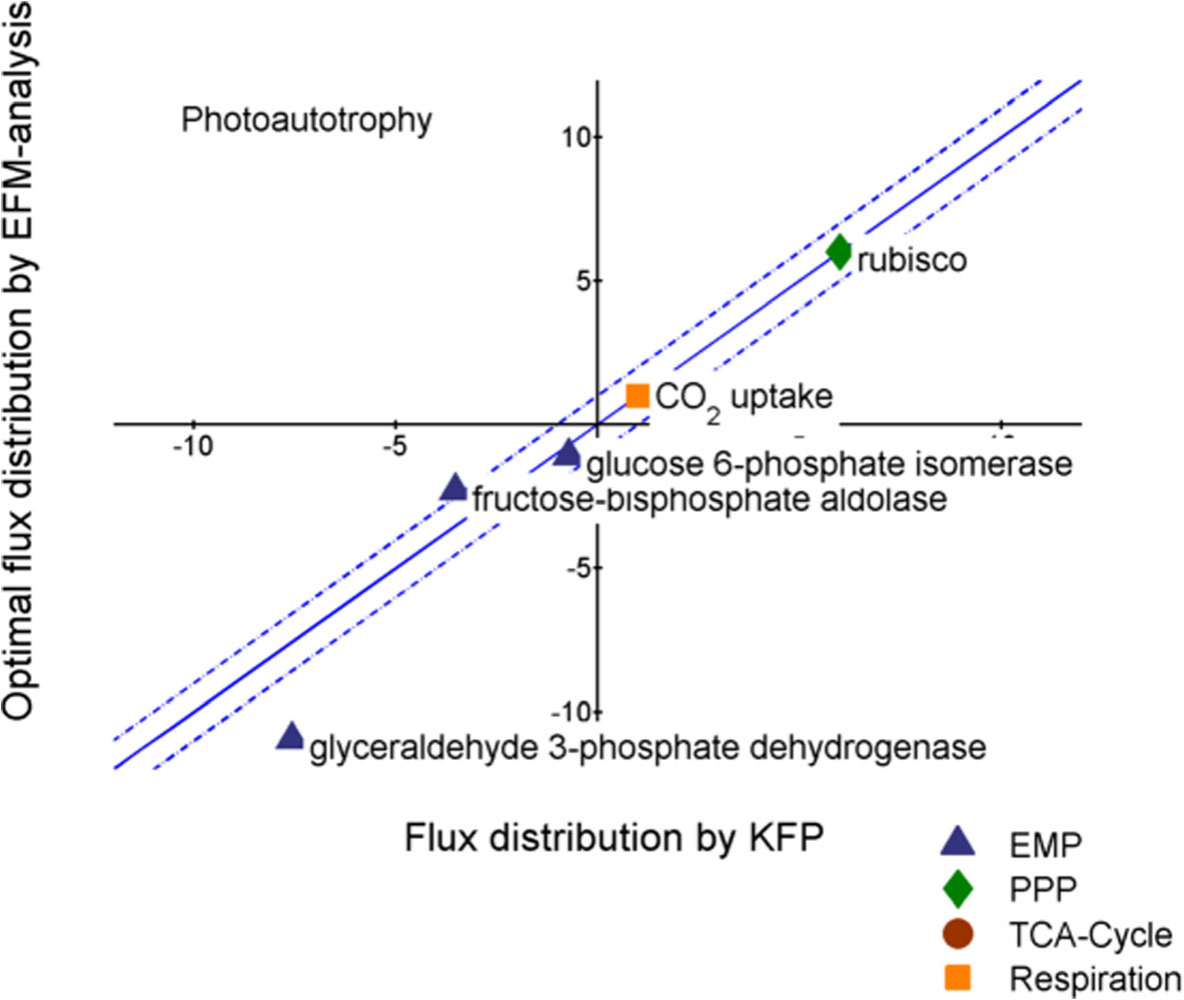
*In silico* versus in vivo metabolic fluxes. Integration of *in silico* with in vivo fluxes: The metabolic phenotype of an illuminated *A. thaliana* rosette, experimentally determined by ^13^C kinetic flux profiling, is integrated with the respective elementary flux mode yielding optimal growth (average flux value of the top 1 % biomass producing modes). All fluxes are expressed as C-mol (6 C-mol substrate)^−1^

The integration of *in silico* and in vivo fluxes allows evaluation of the observed cellular physiology within the overall feasible flux space, as proven valuable for different microorganisms [10, 103]. Fortunately, recent ^13^C flux analysis data from *Arabidopsis* rosettes [38] provide an excellent opportunity to conduct such an integration for the first time in plant cells. For this purpose, the determined flux values were now mapped with the predicted fluxes for optimum growth (Fig. 9). In this context, ‘optimum growth’ refers to the average flux value of the top 1 % biomass producing modes. This provided a striking agreement between the in vivo and optimum *in silico* fluxome for the entire plant. Considering the similarity of measured and predicted biomass and quantum yield, we conclude that illuminated thale cress is represented adequately by the proposed metabolic model, and its leaves operate very close to their maximal potential with regard to stoichiometric capacity. It should be noted that this may not hold for suboptimal growth conditions, such as high light acclimation [100], where the stress conditions seem to induce a metabolic burden. The observed high metabolic efficacy of plants is particularly interesting in the light of their biotechnological potential as only little carbon is ‘wasted’ on respiration and fueling of metabolism. A similar comparison for heterotrophic leaf metabolism could not be drawn due to a lack of experimental data. Unfortunately, whole plant in vivo flux studies in the dark are currently still unattainable [104].

### Synthesis of compositional traits could be driven by dynamic metabolic engineering

Clearly, our simulations indicate that a reversible transhydrogenase-like function is crucial to rearrange the metabolism from day to night (Fig. 2). It is interesting to note that dark and light metabolism involves a rather diverse set of anabolic pathways for optimum precursor supply (Fig. 7). This also involves isoenzymes with different prevalence for NADPH and NADH, which vary between the growth regimes. So far, their role is still unclear, however functional diversification [105] and increased metabolic robustness [106] have been postulated as possible purposes of isoenzymes. Based on our simulations, the ubiquitous presence of isoenzymes with different cofactor usage in plants seems key for flexible handling of specific anabolic demands of day and night metabolism. The extended knowledge on flux re-arrangement between day and night physiologies is particularly interesting for the development of plants with biotechnologically interesting traits. Plants recruit distinct pathways for the synthesis of anabolic precursors and energy during the day and at night (Figs. 6 and 7). This information seems most helpful to optimize precursor, reducing power and energy supply towards biotechnologically interesting traits. On a first glance, constitutive synthesis would permit continuous accumulation of the desired compound. However, biosynthesis might require much more energy or reducing equivalents during the night, as compared to the day. In such cases, it might be more desirable to connect biotechnological syntheses to genes that are tightly regulated throughout the diurnal cycle. Novel approaches, which allow dynamic programming of metabolism [107], seem straightforward to exploit this fundamental plant characteristic.

### Day-night flux rearrangement is superimposed by selected transcriptional changes

**Fig. 10:**
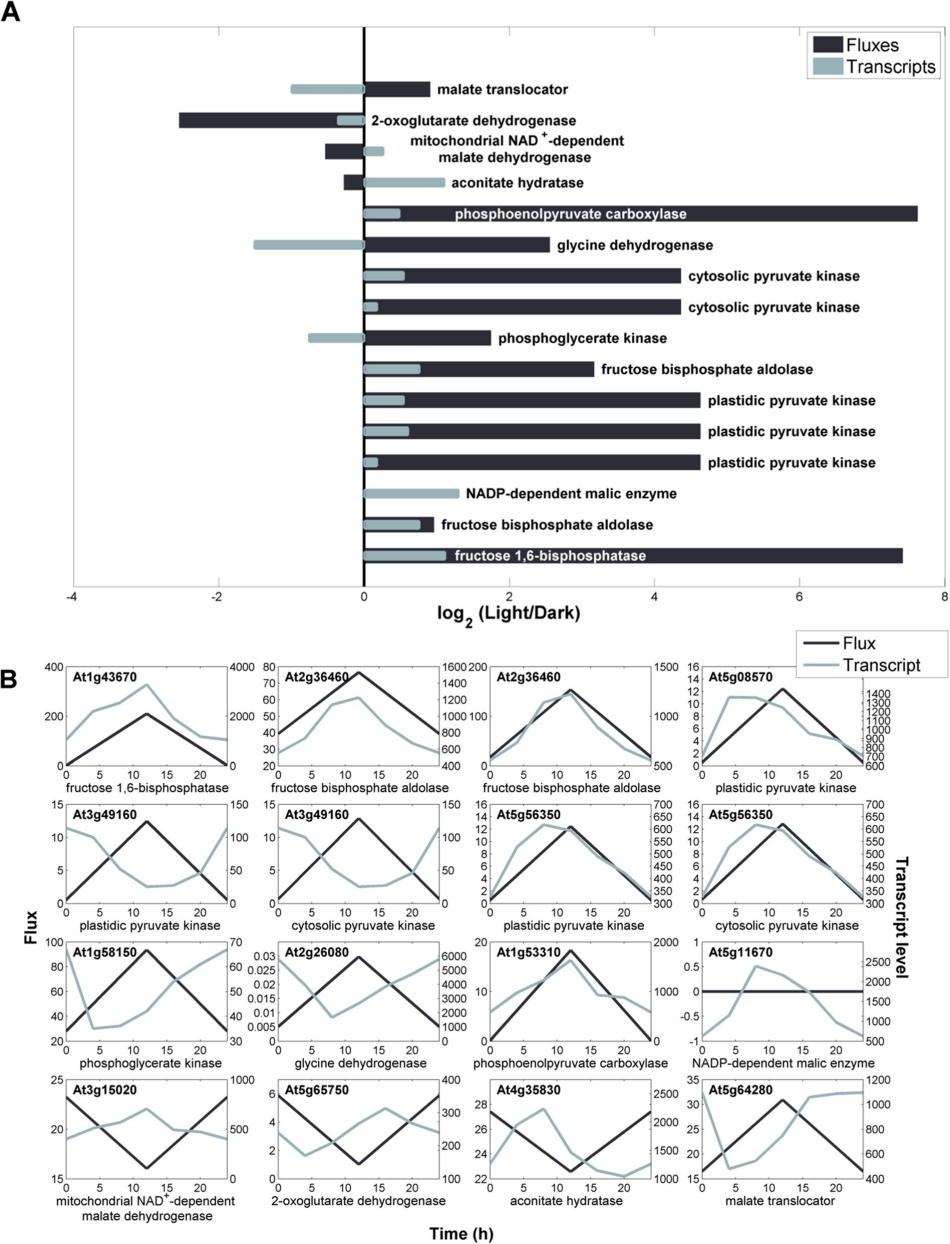
Integration of transcriptome and in silico metabolic fluxes. **a** Comparison of the amplitude in diurnally expressed *A. thaliana* genes [37] and the amplitude in flux change calculated by our model under optimal growth (average flux value of the top 1 % biomass producing modes). **b** Gene expression data are displayed with their expression level, whereas the fluxome is given in C-mol C-mol^−1^. A 12 h light and 12 h dark diurnal cycle was adopted

As shown, the optimum metabolism of *Arabidopsis* recruits a different set of pathways during day and night: diurnal cycling is linked to a substantial redistribution of flux. It was now interesting to see, how regulatory circuits of the plant superimpose the obvious fine-adjustment on the level of the metabolic network, i.e. to which extent transcriptional and post-transcriptional control is involved, and which genes are part of the overarching regulatory network. Our simulation data enabled the integration of fluxes with the expression of encoding genes. This integration of transcriptomic knowledge with the observed flux changes under optimum growth conditions offered a systems level view of the day-night shift (Fig. 10). Reactions, such as the ones catalyzed by mitochondrial and plastidic pyruvate dehydrogenase and citrate synthase were found stable in either fluxome or transcriptome. Others displayed clear diurnal trends, as for instance cytosolic and plastidic pyruvate kinase, phosphoribulokinase, fructose 1,6-bisphosphate phosphatase and many others. Diurnal changes in both transcripts and fluxes were observed for the previously identified on/off switches, i.e. for ribulose 1,5-bisphosphate carboxylase, phosphoribulokinase, fructose 1,6-bisphosphate phosphatase, NADP-dependent glyceraldehyde 3-phosphate dehydrogenase and sedoheptulose 1,7-bisphosphate phosphatase [90]. In addition, the triose phosphate translocator between plastid and cytosol is known to follow a diurnal path for carbon export [90]. This supports the predicted high export flux for 3-phosphoglycerate, phosphoenolpyruvate and dihydroxyacetone phosphate during the light period. Additionally, light/dark modulation of pyruvate-orthophosphate (Pi) dikinase through phosphorylation in C_3_ leaves [108] is confirmed by an off switch during the night. Even for those genes that appear to have unaltered expression levels, although fluxomic changes are observed, isogenes exist that are still to be tested, including reactions of the oxidative PP pathway, fumarase or hexose isomerase. Overall, systems level correlation occurs plentiful and seems well distributed over the entire central carbon metabolism, reflecting the necessity of readjusting the core metabolism during day/night-transition.

### Dynamics in metabolic pathway fluxes and gene expression

A more detailed view on selected transcriptional changes is obtained by overlaying the time resolved expression of metabolic genes from *Arabidopsis* grown with day night cycles [37] with the corresponding flux changes of the encoded reactions, as predicted from the simulations under optimal growth (Fig. 10). In a number of cases, transcription-based and flux-based changes exhibit a close connection, which indicates that the plant strongly recruits regulatory mechanisms to drive core metabolism. Interestingly, the genes that showed analogous behavior in transcript and flux, were located around three main controlling points. Firstly, the fluxes between fructose 6-phosphate and triose-phosphate appeared somewhat regulated. At least, fructose 1,6-bisphosphatase and fructose bisphosphate aldolase showed similar behavior in transcript and flux. Secondly, many enzymes involving malate and pyruvate had a diurnal pattern, indicating malate and/or pyruvate might be a second controlling point in metabolism. Both the fructose 1,6-bisphosphatase and pyruvate kinase controlling points have previously been postulated as diurnal regulators of metabolism [109]. In addition, the complex regulation pattern around the malate node is reflected in the local high network complexity. Finally, genes associated with the TCA cycle seem superimposed by transcriptional regulation.

Besides identifying clusters of potential transcriptional control, this type of analysis can be used to identify, which isoenzymes might be linked to flux changes. For example, for three pyruvate kinase-associated genes (At5g08570, At5g56350 and At3g49160) a diurnal expression pattern was identified, however only the former two are linked to the observed flux changes. This indicates that At3g49160 likely has a functionally different purpose from mediating the pyruvate phosphorylation in central carbon metabolism. For instance, pyruvate kinase could also catalyze several metabolic conversions in ribonucleotide and nucleotide biosynthesis. As isoenzymes might have distinct functions [105], correlation of flux and transcript could pose a valuable method for identification of iso-enzymatic function and might provide first evidence on their biochemical function. This is particularly useful in cases, where a high number of isoenzymes potentially catalyze a certain reaction. Furthermore, flux-transcript correlation can assist in the confirmation of genes with putative function. Putative genes that do not show significant linkage to flux change possibly do not control flux on a transcriptional level (At3g15020, At2g26080, At1g58150, At3g49160, At5g11670). However, those that do correlate might present candidates involved in fructose bisphosphate aldolase (At2g36460), fructose 1,6-bisphosphatase (At1g43670) and phosphoenolpyruvate carboxylase (At1g53310). One should keep in mind for these interpretations, that not in all cases transcript changes will immediately lead to protein change, because translation in plants can be damped or delayed [110].

### Flux-homeostasis and biotechnological impact of plasticity in photophosphorylation

Plants adapt to changes in light intensity through photo protection and optimization of energy conversion [66]. In this way, also the output ratio of ATP: NADPH can be influenced significantly by the light environment, which has been investigated extensively [65–67, 96, 101]. How cellular metabolism copes with the changed ATP: NADPH supply on the level of intracellular fluxes is investigated here. For instance, it seems that the protection of photosystem I against photo-inhibition through an increase in cyclic electron flow, only poses a small metabolic burden, as only little modulation is necessary to deal with the imposed increase in ATP (Fig. 8). The short time frame, in which metabolism needs to react to such sudden fluctuations in light, is also reflected in the fact that the quick onset of large differences in ATP, only requires small flux changes in response. On the short term, these changes might not require modulation of transcript or protein levels, and could therefore warrant an instantaneous short-term response. Additionally, the observed stability of biomass formation, with only few distinct flux changes, across a wide range of ATP: NADPH ratios (Fig. 8), indicates a certain robustness of metabolism to environmental changes. The metabolic fluxes that are most influenced by small changes around the assumed in vivo ratio of non-cyclic and cyclic electron flow of 12:2, involve specific modulation of the NADPH status and of the dissipation of ATP through futile cycling. Possibly, these specific flux changes permit homeostasis of net flux and adenylate/redox status. A homeostasis of the adenylate status during photosynthesis in a fluctuating environment has previously been indicated, however, here, homeostasis was supposedly attributed to changes in pathway usage [111]. Here, we observe that the net flux distribution remains unchanged under abruptly changing light environment, however, strong differences occur in substrate cycling. Such substrate cycling has previously been identified in vivo in plant tissues using experiments [112] and *in silico* through modelling [96]. Likely, the observed metabolic adaptation through futile cycling occurs in addition to changes in pathway use, such as increased photorespiration. This improves the plants’ capacity to cope with a constantly changing light environment, both on the short-term through e.g. ATP dissipation and redox modulation and on the long-term through e.g. changes in pathway usage. Furthermore, it has recently been proposed that the excess in redox power could be directed towards light-driven production of biotechnological compounds through cytochrome P450s-mediated reactions [113]. When an organism is engineered to produce large amounts of a biotechnologically interesting product, its molecular flux patterns change. These metabolic changes engage a different demand for ATP and NADPH that needs to be accustomed by the cell. Due to the observed plasticity in non-cyclic and cyclic electron flow, plants are capable to adapt to such modulated energetic requirements. In addition, our simulations now show that such adaptations do not impede growth, granting plants a high potential for the production of biotechnological products, especially for those compounds requiring much redox power. This emphasizes the potential of photosynthetic light reactions in biotechnology.

## Conclusion

The created condensed network model adequately described leaf physiology, and thus, because of its reduced network size as compared to genome-scale models, permits straightforward, comprehensive analysis of the entire elementary flux mode solution space. Taken together, the metabolic simulations provide detailed molecular insights into plant functioning. *Arabidopsis* can operate close to theoretical pathway optimum and that this is mediated by a fine-adjustment of metabolic flux, strongly under transcriptional control. In this light, the present work is one of the very few examples so far, which link in-vivo with *in-silico* flux data to a higher-level understanding [103, 114, 115]. It seems straightforward to extend this to other plant systems and to more specific models that address specific plant tissues, which are formed during plant development. The knowledge gained from our systems-biological approach, together with the high potential of plants as biotechnological production platforms, especially for compounds requiring much redox power, will contribute to establish plants as biotechnological factories.

## Additional files

Additional file 1: Supplemental tables and figures. (DOCX 1 mb)
Additional file 2: SBML file of full network. (XML 647 kb)
Additional file 3: SBML file of condensed network. (XML 253 kb)
