# Supplementary material for: In silico metabolic network analysis of Arabidopsis leaves
Source: BMC Syst Biol. 2016 Oct 29;10:102. doi: 10.1186/s12918-016-0347-3 (PMC5086045; doi:10.1186/s12918-016-0347-3)
Supplement: Additional file 1: — Supplemental tables and figures. (DOCX 1 mb) [file 12918_2016_347_MOESM1_ESM.docx]

**Supplemental Tables and Figures and Supplemental Information to**

***In silico* metabolic network analysis of *Arabidopsis* leaves**

**Veronique Beckers^1^, Lisa Maria Dersch^1^, Katrin Lotz^2^, Guido Melzer^3^, Oliver Ernst Bläsing^2^, Regine Fuchs^2^, Thomas Ehrhardt^2^, Christoph Wittmann^1§^**

^1^Institute for Systems Biotechnology, Saarland University

^2^Metanomics GmbH

^3^Institute of Biochemical Engineering, Technical University Braunschweig

**^§^**Address of **corresponding author**: Institute for Systems Biotechnology, Saarland University, Campus A1.5, 66123 Saarbrücken, Germany, Phone: +49-681-302-71971, Fax: +49-681-302-71972, Email: [christoph.wittmann@uni-saarland.de](mailto:christoph.wittmann@uni-saarland.de)

**Definitions:**

Biomass Biomass is referred to as the sum of precursors leading to macromolecular building blocks in plant metabolism, normalized to 1 gram of dry matter (defined in S1, S2 and S3).

Growth Growth is defined as the increase in biomass.

**Table S1:** *Concentrations of all biomass components used in the simulations accompanied by their respective source(s).*

|  | Amount  [mg / g DW] | Source |
| --- | --- | --- |
| Carbohydrates   - monosaccharides   - glucose   - fructose   - fucose   - rhamnose   - arabinose   - mannose   - galactose   - xylose   - inositol - disaccharides   - sucrose - polysaccharides   - starch | 9.57  1.60  0.73  0.84  0.53  0.46  6.99  0.33  3.20  16.02  90.70 | ([Tschoep et al., 2009](#_ENREF_13))  ([Tschoep et al., 2009](#_ENREF_13))  ([Reiter et al., 1997](#_ENREF_7))  ([Reiter et al., 1997](#_ENREF_7))  ([Reiter et al., 1997](#_ENREF_7))  ([Reiter et al., 1997](#_ENREF_7))  ([Reiter et al., 1997](#_ENREF_7))  ([Reiter et al., 1997](#_ENREF_7))  ([Saxena et al., 2013](#_ENREF_8))  ([Tschoep et al., 2009](#_ENREF_13))  ([Tschoep et al., 2009](#_ENREF_13)) |
| Cell wall   - cellulose - hemicellulose   - xyloglucan   - glucuronoarabinoxylan - pectin - lignin   - 4-coumaryl-alcohol   - confideryl-alcohol   - sinapyl-alcohol - soluble polymers   - rhamnose   - fucose   - arabinose   - xylose   - mannose   - galactose   - glucose | 46.74  66.77  13.35  140.22  1.38  88.37  48.33  0.17  0.41  2.22  0.49  0.60  4.69  0.15 | ([Reiter et al., 1997](#_ENREF_7))  ([Zablackis et al., 1995](#_ENREF_14))  ([Zablackis et al., 1995](#_ENREF_14)) ([Zablackis et al., 1995](#_ENREF_14))  ([Herrero et al., 2013](#_ENREF_4))  ([Herrero et al., 2013](#_ENREF_4))  ([Herrero et al., 2013](#_ENREF_4))  ([Reiter et al., 1997](#_ENREF_7))  ([Reiter et al., 1997](#_ENREF_7))  ([Reiter et al., 1997](#_ENREF_7))  ([Reiter et al., 1997](#_ENREF_7))  ([Reiter et al., 1997](#_ENREF_7))  ([Reiter et al., 1997](#_ENREF_7))  ([Reiter et al., 1997](#_ENREF_7)) |
| Lipids   - glycerol - fatty acids   - C16:0   - C16:1   - C16:2   - C16:3   - C18:0   - C18:1   - C18:2   - C18:3 | 0.40  5.46  1.41  0.32  4.48  0.48  1.24  5.85  19.22 | ([Fan et al., 2013](#_ENREF_3))  ([Shen et al., 2010](#_ENREF_9); [Stahl et al., 2004](#_ENREF_10))  ([Shen et al., 2010](#_ENREF_9); [Stahl et al., 2004](#_ENREF_10))  ([Shen et al., 2010](#_ENREF_9); [Stahl et al., 2004](#_ENREF_10))  ([Shen et al., 2010](#_ENREF_9); [Stahl et al., 2004](#_ENREF_10))  ([Shen et al., 2010](#_ENREF_9); [Stahl et al., 2004](#_ENREF_10))  ([Shen et al., 2010](#_ENREF_9); [Stahl et al., 2004](#_ENREF_10))  ([Shen et al., 2010](#_ENREF_9); [Stahl et al., 2004](#_ENREF_10))  ([Shen et al., 2010](#_ENREF_9); [Stahl et al., 2004](#_ENREF_10)) |
| Steroids   - sitosterol - stigmasterol | 2.06  0.10 | ([Arnqvist et al., 2008](#_ENREF_1))  ([Arnqvist et al., 2008](#_ENREF_1)) |
| Proteins/Amino Acids   - alanine - arginine - asparagine - aspartate - cysteine - glutamate - glutamine - glycine - histidine - isoleucine - leucine - lysine - methionine - phenylalanine - proline - serine - threonine - tryptophan - tyrosine - valine | 4.34  5.79  12.28  36.25  32.27  87.65  65.60  9.01  0.52  0.46  0.73  0.41  0.40  2.04  7.46  46.28  8.77  0.43  0.29  1.54 | ([Tschoep et al., 2009](#_ENREF_13))  ([Tschoep et al., 2009](#_ENREF_13))  ([Tschoep et al., 2009](#_ENREF_13))  ([Tschoep et al., 2009](#_ENREF_13))  ([de Oliveira Dal'Molin et al., 2010](#_ENREF_2))  ([Tschoep et al., 2009](#_ENREF_13))  ([Tschoep et al., 2009](#_ENREF_13))  ([Tschoep et al., 2009](#_ENREF_13))  ([Tschoep et al., 2009](#_ENREF_13))  ([Tschoep et al., 2009](#_ENREF_13))  ([Tschoep et al., 2009](#_ENREF_13))  ([Tschoep et al., 2009](#_ENREF_13))  ([Tschoep et al., 2009](#_ENREF_13))  ([Tschoep et al., 2009](#_ENREF_13))  ([Tschoep et al., 2009](#_ENREF_13))  ([Tschoep et al., 2009](#_ENREF_13))  ([Tschoep et al., 2009](#_ENREF_13))  ([Tschoep et al., 2009](#_ENREF_13))  ([Tschoep et al., 2009](#_ENREF_13))  ([Tschoep et al., 2009](#_ENREF_13)) |
| Nucleotides   - DNA   - dATP   - dTTP   - dCTP   - dGTP - RNA   - ATP   - UTP   - CTP   - GTP | 1.95  1.95  1.10  1.10  0.22  0.22  0.13  0.13 | ([Suzuki et al., 2004](#_ENREF_11))  ([Suzuki et al., 2004](#_ENREF_11))  ([Suzuki et al., 2004](#_ENREF_11))  ([Suzuki et al., 2004](#_ENREF_11))  ([Murray and Thompson, 1980](#_ENREF_5))  ([Murray and Thompson, 1980](#_ENREF_5))  ([Murray and Thompson, 1980](#_ENREF_5))  ([Murray and Thompson, 1980](#_ENREF_5)) |
| Porphyrines   - chlorophyll   - chlorophyll a   - chlorophyll a - carotenoids   - β-carotene   - zeaxanthin   - lutein   - antheraxanthin   - violaxanthin   - neoxanthin | 3.81  7.78  0.49  0.06  1.07  0.02  0.33  0.26 | ([Nowicka et al., 2009](#_ENREF_6))  ([Nowicka et al., 2009](#_ENREF_6))  ([Tardy and Havaux, 1996](#_ENREF_12))  ([Tardy and Havaux, 1996](#_ENREF_12))  ([Tardy and Havaux, 1996](#_ENREF_12))  ([Tardy and Havaux, 1996](#_ENREF_12))  ([Tardy and Havaux, 1996](#_ENREF_12))  ([Tardy and Havaux, 1996](#_ENREF_12)) |
| Organic acids   - malate - fumarate | 6.76  7.84 | ([Tschoep et al., 2009](#_ENREF_13))  ([Tschoep et al., 2009](#_ENREF_13)) |

**References**

Arnqvist, L., Persson, M., Jonsson, L., Dutta, P. C., Sitbon, F., 2008. Overexpression of CYP710A1 and CYP710A4 in transgenic *Arabidopsis* plants increases the level of stigmasterol at the expense of sitosterol. Planta. 227**,** 309-17.

de Oliveira Dal'Molin, C. G., Quek, L. E., Palfreyman, R. W., Brumbley, S. M., Nielsen, L. K., 2010. AraGEM, a genome-scale reconstruction of the primary metabolic network in Arabidopsis. Plant physiology. 152**,** 579-89.

Fan, J., Yan, C., Zhang, X., Xu, C., 2013. Dual role for phospholipid:diacylglycerol acyltransferase: enhancing fatty acid synthesis and diverting fatty acids from membrane lipids to triacylglycerol in *Arabidopsis* leaves. The Plant cell. 25**,** 3506-18.

Herrero, J., Fernández-Pérez, F., Yebra, T., Novo-Uzal, E., Pomar, F., Pedreño, M. A., Cuello, J., Guéra, A., Esteban-Carrasco, A., Zapata, J. M., 2013. Bioinformatic and functional characterization of the basic peroxidase 72 from *Arabidopsis thaliana* involved in lignin biosynthesis. Planta. 237**,** 1599-612.

Murray, M. G., Thompson, W. F., 1980. Rapid isolation of high molecular weight plant DNA. Nucleic acids research. 8**,** 4321-5.

Nowicka, B., Strzalka, W., Strzalka, K., 2009. New transgenic line of *Arabidopsis thaliana* with partly disabled zeaxanthin epoxidase activity displays changed carotenoid composition, xanthophyll cycle activity and non-photochemical quenching kinetics. Journal of plant physiology. 166**,** 1045-56.

Reiter, W. D., Chapple, C., Somerville, C. R., 1997. Mutants of *Arabidopsis thaliana* with altered cell wall polysaccharide composition. The Plant journal : for cell and molecular biology. 12**,** 335-45.

Saxena, S. C., Salvi, P., Kaur, H., Verma, P., Petla, B. P., Rao, V., Kamble, N., Majee, M., 2013. Differentially expressed *myo*-inositol monophosphatase gene (*CaIMP*) in chickpea (*Cicer arietinum* L.) encodes a lithium-sensitive phosphatase enzyme with broad substrate specificity and improves seed germination and seedling growth under abiotic stresses. Journal of experimental botany. 64**,** 5623-39.

Shen, W., Li, J. Q., Dauk, M., Huang, Y., Periappuram, C., Wei, Y., Zou, J., 2010. Metabolic and transcriptional responses of glycerolipid pathways to a perturbation of glycerol 3-phosphate metabolism in *Arabidopsis*. The Journal of biological chemistry. 285**,** 22957-65.

Stahl, U., Carlsson, A. S., Lenman, M., Dahlqvist, A., Huang, B., Banas, W., Banas, A., Stymne, S., 2004. Cloning and functional characterization of a phospholipid:diacylglycerol acyltransferase from *Arabidopsis*. Plant physiology. 135**,** 1324-35.

Suzuki, Y., Kawazu, T., Koyama, H., 2004. RNA isolation from siliques, dry seeds, and other tissues of *Arabidopsis thaliana*. BioTechniques. 37**,** 542, 544.

Tardy, F., Havaux, M., 1996. Photosynthesis, chlorophyll fluorescence, light-harvesting system and photoinhibition resistance of a zeaxanthin-accumulating mutant of *Arabidopsis thaliana*. Journal of photochemistry and photobiology. B, Biology. 34**,** 87-94.

Tschoep, H., Gibon, Y., Carillo, P., Armengaud, P., Szecowka, M., Nunes-Nesi, A., Fernie, A. R., Koehl, K., Stitt, M., 2009. Adjustment of growth and central metabolism to a mild but sustained nitrogen-limitation in *Arabidopsis*. Plant, cell & environment. 32**,** 300-18.

Zablackis, E., Huang, J., Muller, B., Darvill, A. G., Albersheim, P., 1995. Characterization of the cell-wall polysaccharides of *Arabidopsis thaliana* leaves. Plant physiology. 107**,** 1129-38.

**Table S2:**

*Anabolic precursor demand for biomass synthesis of A. thaliana leaves. Most pathways for synthesis of biomass building blocks are linear and are summarized into a single lumped biomass equation, depending only on a handful of precursors from central carbon metabolism. Information on the organism-specific pathway availability and stoichiometry can be found in the databases specified in the material and methods chapter.*

|  | AcCoA[p] | PYR[c] | CO2[p] | PEP[p] | AKG[m] | R5P[p] | DHAP[p] | E4P[p] | OAA[c] | 3PG[p] | PYR[p] | Pi[c] | Pi[p] | Pi[m] | NADPH[p] | NADPH[c] | NADPH[m] | NAD[p] | NAD[c] | GAP[p] | ATP[p] | ATP[c] | ATP[m] | F6P[c] | G6P[c] | FUM[m] | MAL[c] | STA[p] | GLY[c] | SER[c] |
| --- | --- | --- | --- | --- | --- | --- | --- | --- | --- | --- | --- | --- | --- | --- | --- | --- | --- | --- | --- | --- | --- | --- | --- | --- | --- | --- | --- | --- | --- | --- |
| **Protein** |  |  |  |  |  |  |  |  |  |  |  |  |  |  |  |  |  |  |  |  |  |  |  |  |  |  |  |  |  |  |
| alanine |  | 1 |  |  |  |  |  |  |  |  |  | -2 |  |  | 1 |  |  |  |  |  |  | 2 |  |  |  |  |  |  |  |  |
| arginine |  |  | 1 |  | 1 |  |  |  |  |  |  | -4 |  | -2 |  | 1 | 1 |  |  |  |  | 4 | 3 |  |  |  |  |  |  |  |
| asparagine |  |  |  |  |  |  |  |  | 1 |  |  | -4 | -1 |  |  | 1 |  |  |  |  | 1 | 4 |  |  |  |  |  |  |  |  |
| aspartate |  |  |  |  |  |  |  |  | 1 |  |  | -2 |  |  |  | 1 |  |  |  |  |  | 2 |  |  |  |  |  |  |  |  |
| cysteine |  |  |  |  |  |  |  |  |  | 1 |  |  | -5 |  |  | 5 |  | 1 |  |  | 4 |  |  |  |  |  |  |  |  |  |
| glutamate |  |  |  |  | 1 |  |  |  |  |  |  |  |  | -2 |  |  | 1 |  |  |  |  |  | 2 |  |  |  |  |  |  |  |
| glutamine |  |  |  |  | 1 |  |  |  |  |  |  |  |  | -2 |  |  | 1 |  |  |  |  |  | 3 |  |  |  |  |  |  |  |
| glycine |  |  |  |  |  |  |  |  |  |  |  |  | -2 |  |  |  |  |  |  |  | 2 |  |  |  |  |  |  |  | 1 |  |
| histidine |  |  | 1 |  |  | 1 |  |  |  |  |  |  | -8 |  | 1 |  |  | 2 |  |  | 6 |  |  |  |  |  |  |  |  |  |
| isoleucine |  |  | -1 |  |  |  |  |  | 1 |  | 1 |  | -2 |  | 3 | 1 |  | -1 |  |  | 4 |  |  |  |  |  |  |  |  |  |
| leucine | 1 |  | -2 |  |  |  |  |  |  |  | 2 |  | -2 |  | 2 |  |  | 1 |  |  | 2 |  |  |  |  |  |  |  |  |  |
| lysine |  |  | -1 |  |  |  |  |  | 1 |  | 1 |  | -3 | -1 | 4 |  |  |  |  |  | 3 |  | 1 |  |  |  |  |  |  |  |
| methionine |  |  | 1 |  |  |  |  |  | 1 | 1 | -1 | -2 | -6 |  | 5 | 4 |  | 1 | 2 |  | 7 | 2 |  |  |  |  |  |  |  |  |
| phenylalanine |  |  | -1 | 2 |  |  |  | 1 |  |  |  |  | -6 |  | 2 |  |  |  |  |  | 3 |  |  |  |  |  |  |  |  |  |
| proline |  |  |  |  | 1 |  |  |  |  |  |  | -3 |  |  |  | 2 | 1 |  |  |  |  | 3 | 1 |  |  |  |  |  |  |  |
| serine |  |  |  |  |  |  |  |  |  |  |  |  | -2 |  |  |  |  |  |  |  | 2 |  |  |  |  |  |  |  |  | 1 |
| threonine |  |  |  |  |  |  |  |  | 1 |  |  |  | -4 |  | 1 | 1 |  | 1 |  |  | 4 |  |  |  |  |  |  |  |  |  |
| tryptophan |  |  | -1 | 2 |  | 1 |  | 1 |  | 1 | -1 |  | -9 |  | 2 |  |  | 1 |  | -1 | 6 |  |  |  |  |  |  |  |  |  |
| tyrosine |  |  | -1 | 2 |  |  |  | 1 |  |  |  |  | -6 |  | 2 |  |  | -1 |  |  | 3 |  |  |  |  |  |  |  |  |  |
| valine |  | 2 | -1 |  |  |  |  |  |  |  |  |  | -2 |  | 2 |  |  |  |  |  | 2 |  |  |  |  |  |  |  |  |  |
| **Carbohydrates** |  |  |  |  |  |  |  |  |  |  |  |  |  |  |  |  |  |  |  |  |  |  |  |  |  |  |  |  |  |  |
| glucose |  |  |  |  |  |  |  |  |  |  |  | -2 |  |  |  |  |  |  |  |  |  | 1 |  |  | 1 |  |  |  |  |  |
| fructose |  |  |  |  |  |  |  |  |  |  |  |  |  |  |  |  |  |  |  |  |  | 1 |  | 1 |  |  |  |  |  |  |
| fucose |  |  |  |  |  |  |  |  |  |  |  | -2 |  |  |  | 1 |  |  |  |  |  | 1 |  | 1 |  |  |  |  |  |  |
| inositol |  |  |  |  |  |  |  |  |  |  |  | -1 |  |  |  |  |  |  |  |  |  | 1 |  |  | 1 |  |  |  |  |  |
| rhamnose |  |  |  |  |  |  |  |  |  |  |  | -2 |  |  |  | 1 |  |  |  |  |  | 1 |  |  | 1 |  |  |  |  |  |
| arabinose |  |  | -1 |  |  |  |  |  |  |  |  | -2 |  |  |  |  |  |  | 2 |  |  | 1 |  |  | 1 |  |  |  |  |  |
| mannose |  |  |  |  |  |  |  |  |  |  |  | -2 |  |  |  |  |  |  |  |  |  | 1 |  | 1 |  |  |  |  |  |  |
| galactose |  |  |  |  |  |  |  |  |  |  |  | -2 |  |  |  |  |  |  |  |  |  | 1 |  |  | 1 |  |  |  |  |  |
| xylose |  |  | -1 |  |  |  |  |  |  |  |  | -2 |  |  |  |  |  |  | 2 |  |  | 1 |  |  | 1 |  |  |  |  |  |
| sucrose |  |  |  |  |  |  |  |  |  |  |  | -3 |  |  |  |  |  |  |  |  |  | 1 |  | 1 | 1 |  |  |  |  |  |
| starch |  |  |  |  |  |  |  |  |  |  |  |  |  |  |  |  |  |  |  |  |  |  |  |  |  |  |  | 1 |  |  |
| **Cell wall** |  |  |  |  |  |  |  |  |  |  |  |  |  |  |  |  |  |  |  |  |  |  |  |  |  |  |  |  |  |  |
| cellulose |  |  |  |  |  |  |  |  |  |  |  | -2 |  |  |  |  |  |  |  |  |  | 1 |  |  | 1 |  |  |  |  |  |
| rhamnose |  |  |  |  |  |  |  |  |  |  |  | -2 |  |  |  | 1 |  |  |  |  |  | 1 |  |  | 1 |  |  |  |  |  |
| fucose |  |  |  |  |  |  |  |  |  |  |  | -2 |  |  |  | 1 |  |  |  |  |  | 1 |  | 1 |  |  |  |  |  |  |
| arabinose |  |  | -1 |  |  |  |  |  |  |  |  | -2 |  |  |  |  |  |  | 2 |  |  | 1 |  |  | 1 |  |  |  |  |  |
| xylose |  |  | -1 |  |  |  |  |  |  |  |  | -2 |  |  |  |  |  |  | 2 |  |  | 1 |  |  | 1 |  |  |  |  |  |
| mannose |  |  |  |  |  |  |  |  |  |  |  | -2 |  |  |  |  |  |  |  |  |  | 1 |  | 1 |  |  |  |  |  |  |
| galactose |  |  |  |  |  |  |  |  |  |  |  | -2 |  |  |  |  |  |  |  |  |  | 1 |  |  | 1 |  |  |  |  |  |
| glucose |  |  |  |  |  |  |  |  |  |  |  | -2 |  |  |  |  |  |  |  |  |  | 1 |  |  | 1 |  |  |  |  |  |
| xyloglucan |  |  | -3 |  |  |  |  |  |  |  |  | -14 |  |  |  | 1 |  |  | 6 |  |  | 7 |  | 1 | 6 |  |  |  |  |  |
| glucurono-arabinoxylan |  |  | -2 |  |  |  |  |  |  |  |  | -4 |  |  |  |  |  |  | 4 |  |  | 2 |  |  | 2 |  |  |  |  |  |
| pectin |  |  | 1 |  |  |  |  |  |  |  |  | -2 |  |  |  |  |  |  |  |  |  | 1 |  |  | 1 |  |  |  |  |  |
| **Lignin** |  |  |  |  |  |  |  |  |  |  |  |  |  |  |  |  |  |  |  |  |  |  |  |  |  |  |  |  |  |  |
| 4coumaryl-alcohol |  |  | -1 | 2 |  |  |  | 1 |  |  |  |  | -8 |  | 5 |  |  |  |  |  |  | 5 |  |  |  |  |  |  |  |  |
| confideryl-alcohol |  |  |  | 2 |  |  |  | 1 |  |  |  |  | -8 |  | 6 |  |  |  |  |  |  | 8 |  |  |  |  |  |  |  |  |
| sinapylalcohol |  |  | 1 | 2 |  |  |  | 1 |  |  |  |  | -8 |  | 7 |  |  |  |  |  |  | 6 |  |  |  |  |  |  |  |  |
| **Porphyrines** |  |  |  |  |  |  |  |  |  |  |  |  |  |  |  |  |  |  |  |  |  |  |  |  |  |  |  |  |  |  |
| chlorophyll a |  |  | -9 |  | 8 |  |  |  |  |  | 4 |  | -31 |  | 36 |  |  |  |  | 4 | 29 |  |  |  |  |  |  |  |  |  |
| chlorophyll b |  |  | -9 |  | 8 |  |  |  |  |  | 4 |  | -31 |  | 38 |  |  |  |  | 4 | 29 |  |  |  |  |  |  |  |  |  |
| betacarotene |  |  | -8 |  |  |  |  |  |  |  | 8 |  | -28 |  | 22 |  |  |  |  | 8 | 24 |  |  |  |  |  |  |  |  |  |
| zeaxanthin |  |  | -8 |  |  |  |  |  |  |  | 8 |  | -28 |  | 22 |  |  | -2 |  | 8 | 24 |  |  |  |  |  |  |  |  |  |
| lutein |  |  | -8 |  |  |  |  |  |  |  | 8 |  | -28 |  | 24 |  |  |  |  | 8 | 24 |  |  |  |  |  |  |  |  |  |
| antheraxanthin |  |  | -8 |  |  |  |  |  |  |  | 8 |  | -28 |  | 23 |  |  | -2 |  | 8 | 24 |  |  |  |  |  |  |  |  |  |
| violaxanthin |  |  | -8 |  |  |  |  |  |  |  | 8 |  | -28 |  | 24 |  |  | -2 |  | 8 | 24 |  |  |  |  |  |  |  |  |  |
| neoxanthin |  |  | -8 |  |  |  |  |  |  |  | 8 |  | -28 |  | 24 |  |  | -2 |  | 8 | 24 |  |  |  |  |  |  |  |  |  |
| **Nucleotides** |  |  |  |  |  |  |  |  |  |  |  |  |  |  |  |  |  |  |  |  |  |  |  |  |  |  |  |  |  |  |
| ATP |  |  | 2 |  |  | 1 |  |  |  | 1 |  |  |  |  | 2 |  |  | 2 |  |  | 13 |  |  |  |  |  |  |  |  |  |
| UTP |  |  |  |  |  | 1 |  |  | 1 |  |  |  |  |  | 1 |  |  |  |  |  | 5 |  |  |  |  |  |  |  |  |  |
| CTP |  |  |  |  |  | 1 |  |  | 1 |  |  |  |  |  | 1 |  |  |  |  |  | 6 |  |  |  |  |  |  |  |  |  |
| GTP |  |  | 2 |  |  | 1 |  |  |  | 1 |  |  |  |  | 2 |  |  | 3 |  |  | 16 |  |  |  |  |  |  |  |  |  |
| dATP |  |  | 2 |  |  | 1 |  |  |  | 1 |  |  |  |  | 3 |  |  | 2 |  |  | 13 |  |  |  |  |  |  |  |  |  |
| dTTP |  |  | 1 |  |  | 1 |  |  | 1 |  |  |  |  |  | 3 |  |  |  |  |  | 8 |  |  |  |  |  |  |  |  |  |
| dCTP |  |  |  |  |  | 1 |  |  | 1 |  |  |  |  |  | 2 |  |  |  |  |  | 6 |  |  |  |  |  |  |  |  |  |
| dGTP |  |  | 2 |  |  | 1 |  |  |  | 1 |  |  |  |  | 3 |  |  | 3 |  |  | 16 |  |  |  |  |  |  |  |  |  |
| **Lipids** |  |  |  |  |  |  |  |  |  |  |  |  |  |  |  |  |  |  |  |  |  |  |  |  |  |  |  |  |  |  |
| C16:0 | 8 |  |  |  |  |  |  |  |  |  |  |  | -8 |  | 7 |  |  | -7 |  |  | 8 |  |  |  |  |  |  |  |  |  |
| C16:1 | 8 |  |  |  |  |  |  |  |  |  |  |  | -8 |  | 8 |  |  | -7 |  |  | 8 |  |  |  |  |  |  |  |  |  |
| C16:2 | 8 |  |  |  |  |  |  |  |  |  |  |  | -8 |  | 8 |  |  | -7 |  |  | 8 |  |  |  |  |  |  |  |  |  |
| C16:3 | 8 |  |  |  |  |  |  |  |  |  |  |  | -8 |  | 9 |  |  | -7 |  |  | 8 |  |  |  |  |  |  |  |  |  |
| C18:0 | 9 |  |  |  |  |  |  |  |  |  |  |  | -9 |  | 8 |  |  | -8 |  |  | 9 |  |  |  |  |  |  |  |  |  |
| C18:1 | 9 |  |  |  |  |  |  |  |  |  |  |  | -9 |  | 9 |  |  | -8 |  |  | 9 |  |  |  |  |  |  |  |  |  |
| C18:2 | 9 |  |  |  |  |  |  |  |  |  |  |  | -9 |  | 9 |  |  | -8 |  |  | 9 |  |  |  |  |  |  |  |  |  |
| C18:3 | 9 |  |  |  |  |  |  |  |  |  |  |  | -9 |  | 10 |  |  | -8 |  |  | 9 |  |  |  |  |  |  |  |  |  |
| Glycerol |  |  |  |  |  |  | 1 |  |  |  |  |  |  |  | 1 |  |  |  |  |  |  |  |  |  |  |  |  |  |  |  |
| **Organic acids** |  |  |  |  |  |  |  |  |  |  |  |  |  |  |  |  |  |  |  |  |  |  |  |  |  |  |  |  |  |  |
| malate |  |  |  |  |  |  |  |  |  |  |  |  |  |  |  |  |  |  |  |  |  |  |  |  |  |  | 1 |  |  |  |
| fumarate |  |  |  |  |  |  |  |  |  |  |  |  |  |  |  |  |  |  |  |  |  |  |  |  |  | 1 |  |  |  |  |
| **Stereoids** |  |  |  |  |  |  |  |  |  |  |  |  |  |  |  |  |  |  |  |  |  |  |  |  |  |  |  |  |  |  |
| sitosterol |  |  | -7 |  |  |  |  |  |  |  | 6 |  | -18 |  | 25 |  |  | -4 |  | 6 | 18 |  |  |  |  |  |  |  |  |  |
| stigmasterol |  |  | -7 |  |  |  |  |  |  |  | 6 |  | -18 |  | 26 |  |  | -4 |  | 6 | 18 |  |  |  |  |  |  |  |  |  |

**Table S3:**

*Reaction Network for* Arabidopsis thaliana *leaf metabolism. Abbreviations can be found in supplemental Table S4*

| **Reaction number** | **Reaction** |
| --- | --- |
| **1 2 3 4 5** | ***In silico* transport reactions**  'BM[c] -->' '--> CO2EX[p]' '--> STA[p]' 'CO2[cel]-->' ' <==> Pi[v]' |
| **6** | **Biomass Synthesis**  '(1.238) ACCOA[p] + (0.075) PYR[c] + (0.360) CO2[cel] + (0.820) PEP[p] + (1.245) AKG[m] + (0.019) R5P[p] + (0.004) DHAP[p] + (0.410) E4P[p] + (0.455) OAA[c] + 0.278 3PG[p] + (0.126) PYR[p] + (2.206) NADPH[p] + (4.513) NADPH[c] + (1.143) NADPH[m] + (0.899) NADH[p] + (0.619) NAD[c] + (0.114) GAP[p] + (0.132) F6P[c] + (1.703) G6P[c] + (0.2652) STA[p] + (0.068) FUM[m] + (0.050) MAL[c] + 0.440 SER[c] + 0.120 GLY[c] + (4.652) ATP[p] + (6.013) ATP[c] + (2.705) ATP[m] --> BM[c] + (2.206) NADP[p] + (4.513) NADP[c] + (1.143) NADP[m] + (0.899) NAD[p] + (0.619) NADH[c] + (4.652) ADP[p] + (6.013) ADP[c] + (2.705) ADP[m] + (4.933) Pi[c] + (8.023) Pi[p] + (2.158) Pi[m]' |
| **7 8 9 10 11 12 13 14 15 16 17 18**  **19 20 21 22**  **23 24 25 26 27 28 29 30**  **31 32 33 34 35 36 37**  **38 39**  **40 41 42 43 44** | **Plastidic Metabolism**  'G6P[p] <==> F6P[p]' 'F6P[p] + ATP[p] --> FBP[p] + ADP[p]' 'FBP[p] --> F6P[p] + Pi[p]'  'FBP[p] <==> DHAP[p] + GAP[p]' 'DHAP[p] <==> GAP[p]' 'GAP[p] + NADP[p] + Pi[p] <==> NADPH[p] + 13bPG[p]' '13bPG[p] + ADP[p] <==> ATP[p] + 3PG[p]' '3PG[p] <==> 2PG[p]' '2PG[p] <==> PEP[p]' 'PEP[p] + ADP[p] --> PYR[p] + ATP[p]'  'PYR[p] + ATP[p] --> PEP[p] + AMP[p] + Pi[p]' 'AMP[p] + ATP[p] <==> 2 ADP[p]'  'PYR[p] + E2Pr-lip[p] --> E2Pr-acet-lip[p] + CO2[cel]' 'E2Pr-acet-lip[p] --> ACCOA[p] + E2Pr-2hyd-lip[p]' 'E2Pr-2hyd-lip[p] + NAD[p] --> E2Pr-lip[p] + NADH[p]' 'MAL[p] + NADP[p] --> NADPH[p] + CO2[cel] + PYR[p]'  'G6P[p] + NADP[p] --> NADPH[p] + 6PGL[p]' '6PGL[p] --> 6PG[p]' '6PG[p] + NADP[p] --> NADPH[p] + RU5P[p] + CO2[cel]' 'R5P[p] <==> RU5P[p]' 'RU5P[p] <==> XU5P[p]' 'GAP[p] + S7P[p] <==> E4P[p] + F6P[p]' 'E4P[p] + XU5P[p] <==> F6P[p] + GAP[p]' 'S7P[p] + GAP[p] <==> R5P[p] + XU5P[p]'  'RU5P[p] + ATP[p] --> RBP[p] + ADP[p]' 'CO2EX[p] + RBP[p] --> (2) 3PG[p]' 'RBP[p] --> 2PGO[p] + 3PG[p]'  '2PGO[p] --> GLYCO[p] + Pi[p]' 'DHAP[p] + E4P[p] --> SBP[p]' 'SBP[p] --> S7P[p] + Pi[p]' 'GLYCER[p] + ATP[p] --> 3PG[p] + ADP[p]'  'MAL[p] + NAD[p] <==> OAA[p] + NADH[p]'  'MAL[p] + NADP[p] <==> OAA[p] + NADPH[p]'  'STA[p] --> MALT[p]' 'G6P[p] <==> aG6P[p]' 'aG6P[p] <==> G1P[p]' 'G1P[p] + ATP[p] --> ADP-GLC[p] + 2 Pi[p]' '2 ADP-GLC[p] --> 2 ADP[p] + STA[p]' |
| **45 46 47 48 49 50 51 52 53 54 55 56**  **57 58 59 60 61**  **62 63 64 65 66 67** | **Cytosolic Metabolism**  'G6P[c] <==> F6P[c]' 'F6P[c] + ATP[c] <==> FBP[c] + ADP[c]' 'FBP[c] <==> DHAP[c] + GAP[c]' 'GAP[c] <==> DHAP[c]'  'GAP[c] + NADP[c] --> 3PG[c] + NADPH[c]'  'GAP[c] + NAD[c] + Pi[c] <==> 13dPG[c] + NADH[c]'   '13dPG[c] + ADP[c] <==> ATP[c] + 3PG[c]' '3PG[c] <==> 2PG[c]' '2PG[c] <==> PEP[c]' 'PYR[c] + ATP[c] --> PEP[c] + AMP[c] + Pi[c]' 'AMP[c] + ATP[c] <==> 2 ADP[c]'  'PEP[c] + ADP[c] --> PYR[c] + ATP[c]'  'G6P[c] + NADP[c] --> NADPH[c] + 6PGL[c]' '6PGL[c] --> 6PG[c]' '6PG[c] + NADP[c] --> NADPH[c] + RU5P[c] + CO2[cel]'  'RU5P[c] <==> XU5P[c]' 'RU5P[c] <==> R5P[c]'  'OAA[c] + ATP[c] --> CO2[cel] + PEP[c] + ADP[c]' 'PEP[c] + CO2[cel] --> OAA[c] + Pi[c]' 'CIT[c] + ATP[c] --> ACCOA[c] + OAA[c] + ADP[c] + Pi[c]' 'MAL[c] + NAD[c] <==> OAA[c] + NADH[c]' 'MALT[c] --> 2 GLC[c]' 'GLC[c] + ATP[c] --> ADP[c] + G6P[c]' |
| **68 69 70 71 72** | **Perixosomal metabolism**  'GLYCO[pe] --> GLYOX[pe]' 'GLYOX[pe] + NADH[pe] --> GLY[pe] + NAD[pe]' 'SER[pe] + GLYOX[pe] --> GLY[pe] + HPYR[pe]' 'HPYR[pe] + NADH[pe] --> NAD[pe] + GLYCER[pe]' 'MAL[pe] + NAD[pe] <==> OAA[pe] + NADH[pe]' |
| **73 74 75**  **76 77 78 79 80 81 82 83 84 85 86 87**  **88 89 90 91** | **Mitochondrial metabolism**  'PYR[m] + E2Pr-lip[m] --> E2Pr-acet-lip[m] + CO2[cel]' 'E2Pr-acet-lip[m] --> ACCOA[m] + E2Pr-2hyd-lip[m]' 'E2Pr-2hyd-lip[m] + NAD[m] --> E2Pr-lip[m] + NADH[m]'  'ACCOA[m] + OAA[m] --> CIT[m]' 'CIT[m] <==> ACO[m]' 'ACO[m] <==> ICIT[m]' 'ICIT[m] + NAD[m] <==> AKG[m] + CO2[cel] + NADH[m]' 'ICIT[m] + NADP[m] <==> AKG[m] + CO2[cel] + NADPH[m]' 'AKG[m] + NAD[m] --> SUCCCOA[m] + CO2[cel] + NADH[m]' 'SUCCCOA[m] + ADP[m] + Pi[m] <==> SUCC[m] + ATP[m]' 'SUCC[m] + UQN[m] <==> FUM[m] + UQL[m]' 'UQL[m] + NAD[m] <==> UQN[m] + NADH[m]' 'FUM[m] --> MAL[m]' 'MAL[m] + NAD[m] --> OAA[m] + NADH[m]' 'MAL[m] + NAD[m] --> CO2[cel] + NADH[m] + PYR[m]'  'GLY[m] + MTHF[m] <==> THF[m] + SER[m]' 'GLY[m] + LP[m] <==> CO2[cel] + SADHLP[m]' 'SADHLP[m] + THF[m] <==> MTHF[m] + DLP[m]' 'DLP[m] + NAD[m] <==> NADH[m] + LP[m]' |
| **92 93 94 95 96 97 98 99**  **100 101 102 103 104 105**  **106 107 108 109 110 111 112 113**  **114 115 116 117 118** | **Transporters**  'GLYCO[p] <==> GLYCO[c]' 'GLYCO[c] <==> GLYCO[pe]' 'GLY[pe] <==> GLY[c]' 'GLY[c] <==> GLY[m]' 'SER[m] <==> SER[c]' 'SER[c] <==> SER[pe]' 'GLYCER[pe] <==> GLYCER[c]' 'GLYCER[c] <==> GLYCER[p]'  'MALT[p] --> MALT[c]' 'G6P[p] + Pi[c] <==> G6P[c] + Pi[p]'  'XU5P[p] + Pi[c] <==> XU5P[c] + Pi[p]'  '3PG[p] + Pi[c] <==> 3PG[c] + Pi[p]'  'DHAP[c] + Pi[p] <==> DHAP[p] + Pi[c] ' 'PEP[c] + Pi[p] <==> PEP[p] + Pi[c]'  'PYR[p] <==> PYR[c]'  'PYR[c] <==> PYR[m]' 'MAL[c] --> MAL[pe]' 'OAA[pe] --> OAA[c]' 'MAL[c] + Pi[m] <==> MAL[m] + Pi[c]'  'OAA[c] + MAL[m] <==> MAL[c] + OAA[m]' 'OAA[c] + MAL[p] <==> MAL[c] + OAA[p]' 'OAA[c] + CIT[m] <==> CIT[c] + OAA[m]'  'Pi[c] --> Pi[m]' 'Pi[c] <==> Pi[p]' 'Pi[v] <==> Pi[c]' 'ATP[m] + ADP[c] --> ADP[m] + ATP[c]' 'ATP[p] + ADP[c] <==> ADP[p] + ATP[c]' |
| **119 120 121 122 123 124 125**  **126 127 128**  **129** | **Energy metabolism**  '2 Hv[p] + PQN[p] --> PQL[p] + 2 H_nc[p]' 'PQL[p] + 2 PC_o[p] <==> PQN[p] + 2 PC_r[p] + 2 H_nc[p]' 'Hv[p] + PC_r[p] + FE_o[p] --> PC_o[p] + FE_r[p]' '2 FE_r[p] + NADP[p] --> 2 FE_o[p] + NADPH[p] + 2 H_nc[p]' 'Hv[p] --> 2 H_c[p]' ' --> Hv[p]'  '2 H_c[p] + 12 H_nc[p] + 3 ADP[p] --> 3 ATP[p]'  'ATP[p] --> ADP[p] + ATP_maint[cel]' '(2.4) ADP[m] + NADH[m] + 2.4 Pi[m] --> NAD[m] + (2.4) ATP[m]' ' ATP_maint[cel] --> '  'NADP[c] + NADH[c] <==> NADPH[c] + NAD[c]' |

**Table S4:**

*Abbreviations used in Table S3*

| **Abbreviation** | **metabolite name** | **Abbreviation** | **metabolite name** |
| --- | --- | --- | --- |
| 13bPG | 1,3-bisphosphoglycerate | GLYOX | glyoxylate |
| 2PG | 2-phosphoglycerate | H_c | H^+^ produced through cyclic photophosphorylation |
| 2PGO | 2-phosphoglycolate | H_nc | H^+^ produced through non-cyclic photophosphorylation |
| 3PG | 3-phosphoglycerate | HPYR | hydroxypyruvate |
| 6PG | gluconate 6-phosphate | Hv | photon |
| 6PGL | 6-phospho glucono-1,5-lactone | ICIT | isocitrate |
| ACCOA | acetyl-CoA | LP | lipoylprotein |
| ACO | cis-aconitate | MAL | malate |
| ADP | adenosine diphosphate | MALT | maltose |
| ADP-GLC | ADP-glucose | MTHF | 5-methyltetrahydrofolate |
| aG6P | α-glucose 6-phosphate | NAD | nicotinamide adenine dinucleotide (oxidized) |
| AKG | α-ketoglutarate | NADH | nicotinamide adenine dinucleotide (reduced) |
| AMP | adenosine monophosphate | NADP | nicotinamide adenine dinucleotide phosphate (oxidized) |
| ATP | adenosine triphosphate | NADPH | nicotinamide adenine dinucleotide phosphate (reduced) |
| ATP_maint | maintenance ATP | OAA | oxaloacetate |
| BM | biomass | PC_o | plastocyanin (oxidized) |
| CIT | citrate | PC_r | plastocyanin (reduced) |
| CO2 | internal CO_2_ | PEP | phosphoenolpyruvate |
| CO2EX | assimilated CO_2_ | Pi | phosphate |
| DHAP | dihydroxyacetone phosphate | PQL | plastoquinol |
| DLP | dihydrolipoylprotein | PQN | plastoquinone |
| E2Pr-2hyd-lip | [pyruvate dehydrogenase E2 protein]- dihydrolipoyl-L-lysine | PYR | pyruvate |
| E2Pr-acet-lip | [pyruvate dehydrogenase E2 protein]-S-acetyldihydrolipoyl-L-lysine | R5P | ribose 5-phosphate |
| E2Pr-lip | [pyruvate dehydrogenase E2 protein]-lipoyl-L-lysine | RBP | ribulose 1.5-bisphosphate |
| E4P | erythrose 4-phosphate | RU5P | ribulose 5-phosphate |
| F6P | fructose 6-phosphate | S7P | sedoheptulose 7-phosphate |
| FBP | fructose 1,6-bisphosphate | SADHLP | S-aminomethyl-dihydrolipoylprotein |
| FE_o | ferredoxin (oxidized) | SBP | sedoheptulose 1,7-bisphosphate |
| FE_r | ferredoxin (reduced) | SER | serine |
| FUM | fumarate | STA | starch |
| G1P | glucose 1-phosphate | SUCC | succinate |
| G6P | glucose 6-phosphate | SUCCCOA | succinyl-CoA |
| GAP | glyceraldehyde 3-phosphate | THF | tetrahydrofolate |
| GLC | glucose | UQL | ubiquinol |
| GLY | glycine | UQN | ubiquinone |
| GLYCER | glycerate | XU5P | xylulose 5-phosphate |
| GLYCO | glycolate |  |  |

**Table S5:**

*Information on reversibility was gathered from Aracyc, KEGG and Metacrop. Y: reaction is annotated as reversible, N: reaction is considered irreversible. Only those reactions for which the information on reversibility was derived from the above-mentioned databases are listed. The remaining reaction reversibilities either originated from the inherent network structure (for uptake and synthesis reactions) or were extracted from the literature as described earlier (transport reactions and energy metabolism).*

| Aracyc | KEGG | MetaCrop | ID | Reaction |
| --- | --- | --- | --- | --- |
| Y | Y | Y | r7 | G6P[p] <==> F6P[p] |
| N | N | N | r8 | F6P[p] + ATP[p] --> FBP[p] + ADP[p] |
| N | N | N | r9 | FBP[p] --> F6P[p] + Pi[p] |
| Y | Y | Y | r10 | FBP[p] <==> DHAP[p] + GAP[p] |
| Y | Y | Y | r11 | DHAP[p] <==> GAP[p] |
| Y | Y | Y | r12 | GAP[p] + NADP[p] + Pi[p] <==> NADPH[p] + 13bPG[p] |
| Y | Y | Y | r13 | 13bPG[p] + ADP[p] <==> ATP[p] + 3PG[p] |
| Y | Y | Y | r14 | 3PG[p] <==> 2PG[p] |
| Y | Y | Y | r15 | 2PG[p] <==> PEP[p] |
| N | N | N | r16 | PEP[p] + ADP[p] --> PYR[p] + ATP[p] |
| N | N | Y | r17 | PYR[p] + ATP[p] --> PEP[p] + AMP[p] + Pi[p] |
| N | Y | Y | r18 | AMP[p] + ATP[p] <==> 2 ADP[p] |
| N | N | N | r19 | PYR[p] + E2Pr-lip[p] --> E2Pr-acet-lip[p] + CO2[cel] |
| N | Y | N | r20 | E2Pr-acet-lip[p] --> ACCOA[p] + E2Pr-2hyd-lip[p] |
| N | Y | N | r21 | E2Pr-2hyd-lip[p] + NAD[p] --> E2Pr-lip[p] + NADH[p] |
| N | N | Y | r22 | MAL[p] + NADP[p] --> NADPH[p] + CO2[cel] + PYR[p] |
| N | N | Y | r23 | G6P[p] + NADP[p] --> NADPH[p] + 6PGL[p] |
| N | N | N | r24 | 6PGL[p] --> 6PG[p] |
| N | N | N | r25 | 6PG[p] + NADP[p] --> NADPH[p] + RU5P[p] + CO2[cel] |
| Y | Y | Y | r26 | R5P[p] <==> RU5P[p] |
| Y | Y | Y | r27 | RU5P[p] <==> XU5P[p] |
| Y | Y | Y | r28 | GAP[p] + S7P[p] <==> E4P[p] + F6P[p] |
| Y | Y | Y | r29 | E4P[p] + XU5P[p] <==> F6P[p] + GAP[p] |
| Y | Y | Y | r30 | S7P[p] + GAP[p] <==> R5P[p] + XU5P[p] |
| N | N | N | r31 | RU5P[p] + ATP[p] --> RBP[p] + ADP[p] |
| N | N | Y | r32 | CO2EX[p] + RBP[p] --> (2) 3PG[p] |
| N | N | N | r33 | RBP[p] --> 2PGO[p] + 3PG[p] |
| N | N | N | r34 | 2PGO[p] --> GLYCO[p] + Pi[p] |
| N | N | Y | r35 | DHAP[p] + E4P[p] --> SBP[p] |
| N | N | N | r36 | SBP[p] --> S7P[p] + Pi[p] |
| N | N | N | r37 | GLYCER[p] + ATP[p] --> 3PG[p] + ADP[p] |
| Y | Y | Y | r38 | MAL[p] + NAD[p] <==> OAA[p] + NADH[p] |
| Y | Y | Y | r39 | MAL[p] + NADP[p] <==> OAA[p] + NADPH[p] |
| N | N | N | r40 | STA[p] --> MALT[p] |
| Y | Y |  | r41 | G6P[p] <==> aG6P[p] |
| Y | Y | Y | r42 | aG6P[p] <==> G1P[p] |
| N | N | Y | r43 | G1P[p] + ATP[p] --> ADP-GLC[p] + 2 Pi[p] |
| N | N | N | r44 | 2 ADP-GLC[p] --> 2 ADP[p] + STA[p] |
| Y | Y | Y | r45 | G6P[c] <==> F6P[c] |
| Y | Y | Y | r46 | F6P[c] + ATP[c] <==> FBP[c] + ADP[c] |
| Y | Y | Y | r47 | FBP[c] <==> DHAP[c] + GAP[c] |
| Y | Y | Y | r48 | GAP[c] <==> DHAP[c] |
| N | N | N | r49 | GAP[c] + NADP[c] --> 3PG[c] + NADPH[c] |
| Y | Y | Y | r50 | GAP[c] + NAD[c] + Pi[c] <==> 13dPG[c] + NADH[c] |
| Y | Y | Y | r51 | 13dPG[c] + ADP[c] <==> ATP[c] + 3PG[c] |
| Y | Y | Y | r52 | 3PG[c] <==> 2PG[c] |
| Y | Y | Y | r53 | 2PG[c] <==> PEP[c] |
| N | N | Y | r54 | PYR[c] + ATP[c] --> PEP[c] + AMP[c] + Pi[c] |
| N | Y | Y | r55 | AMP[c] + ATP[c] <==> 2 ADP[c] |
| N | N | N | r56 | PEP[c] + ADP[c] --> PYR[c] + ATP[c] |
| N | N | Y | r57 | G6P[c] + NADP[c] --> NADPH[c] + 6PGL[c] |
| N | N | N | r58 | 6PGL[c] --> 6PG[c] |
| N | N | N | r59 | 6PG[c] + NADP[c] --> NADPH[c] + RU5P[c] + CO2[cel] |
| Y | Y | Y | r60 | RU5P[c] <==> XU5P[c] |
| Y | Y | Y | r61 | RU5P[c] <==> R5P[c] |
| N | N | N | r62 | OAA[c] + ATP[c] --> CO2[cel] + PEP[c] + ADP[c] |
| Y | N | N | r63 | PEP[c] + CO2[cel] --> OAA[c] + Pi[c] |
| N | Y | N | r64 | CIT[c] + ATP[c] --> ACCOA[c] + OAA[c] + ADP[c] + Pi[c] |
| Y | Y | Y | r65 | MAL[c] + NAD[c] <==> OAA[c] + NADH[c] |
| N | N |  | r66 | MALT[c] --> 2 GLC[c] |
| N | N | N | r67 | GLC[c] + ATP[c] --> ADP[c] + G6P[c] |
| N | N | N | r68 | GLYCO[pe] --> GLYOX[pe] |
| Y | N | N | r69 | GLYOX[pe] + NADH[pe] --> GLY[pe] + NAD[pe] |
| Y | N | N | r70 | SER[pe] + GLYOX[pe] --> GLY[pe] + HPYR[pe] |
| N | N | N | r71 | HPYR[pe] + NADH[pe] --> NAD[pe] + GLYCER[pe] |
| Y | Y | Y | r72 | MAL[pe] + NAD[pe] <==> OAA[pe] + NADH[pe] |
| N | N | N | r73 | PYR[m] + E2Pr-lip[m] --> E2Pr-acet-lip[m] + CO2[cel] |
| N | N | N | r74 | E2Pr-acet-lip[m] --> ACCOA[m] + E2Pr-2hyd-lip[m] |
| N | Y | N | r75 | E2Pr-2hyd-lip[m] + NAD[m] --> E2Pr-lip[m] + NADH[m] |
| N | N | N | r76 | ACCOA[m] + OAA[m] --> CIT[m] |
| Y | Y | Y | r77 | CIT[m] <==> ACO[m] |
| Y | Y | Y | r78 | ACO[m] <==> ICIT[m] |
| N | Y | Y | r79 | ICIT[m] + NAD[m] <==> AKG[m] + CO2[cel] + NADH[m] |
| N | Y | Y | r80 | ICIT[m] + NADP[m] <==> AKG[m] + CO2[cel] + NADPH[m] |
| N | N | N | r81 | AKG[m] + NAD[m] --> SUCCCOA[m] + CO2[cel] + NADH[m] |
| Y | Y | Y | r82 | SUCCCOA[m] + ADP[m] + Pi[m] <==> SUCC[m] + ATP[m] |
| N | Y | Y | r83 | SUCC[m] + UQN[m] <==> FUM[m] + UQL[m] |
| Y | Y | Y | r84 | UQL[m] + NAD[m] <==> UQN[m] + NADH[m] |
| N | Y | N | r85 | FUM[m] --> MAL[m] |
| N | Y | N | r87 | MAL[m] + NAD[m] --> CO2[cel] + NADH[m] + PYR[m] |
| N | Y | Y | r88 | GLY[m] + MTHF[m] <==> THF[m] + SER[m] |
| Y | N | Y | r89 | GLY[m] + LP[m] <==> CO2[cel] + SADHLP[m] |
| Y | N | Y | r90 | SADHLP[m] + THF[m] <==> MTHF[m] + DLP[m] |
| Y | N | Y | r91 | DLP[m] + NAD[m] <==> NADH[m] + LP[m] |

**Table S6:**

*Supplemental Data to Sulpice et al., 2013 with an additional column for the calculated biomass yield.* $Calculated Biomass Yield daytime=\frac{RGR \cdot\frac{DRY MASS}{100}}{net C assimilation \cdot\frac{1 mol C}{{10}^{6}\mu mol C}}$

| photo-period | RGR | DRY MASS | net C assimilation per 24 h cycle | Calculated Biomass Yield daytime |
| --- | --- | --- | --- | --- |
| *h of light* | ***g FW (g FW * d)^-1^*** | ***% FW*** | ***µmol C ( g FW * d)^-1^*** | ***g DW (mol C)^-1^*** |
| 6 | 0.11351 | 8.94 | 420 | 24.2 |
| 8 | 0.17081 | 8.74 | 641 | 23.3 |
| 12 | 0.26000 | 9.14 | 944 | 25.2 |
| 18 | 0.3065 | 8.95 | 972 | 28.2 |

**Table S7:**

*Flux changes between dark and light metabolism. The averaged flux value during both the light and the dark phase have been calculated based on the top 1% biomass producing modes. If the absolute value of the log2-value of the ratio between averaged flux in the light and averaged flux in the dark is larger than 0.5 and if a statistical two-sample t-test assuming unequal variance on the optimal autotrophic and optimal heterotrophic modes was accepted (value = 1), based on a p-value < 0.05, the flux changes between dark and light metabolism were considered significant. P-values that were smaller than 1E-250 were rounded to 0.*

| Reaction | Averaged flux value during  autotrophy [C-mol C-mol^-1^] | Averaged flux value during heterotrophy  [C-mol C-mol^-1^] | log_2_(Light/Dark) | ttest hyptohesis accepted? | pvalue ttest | significant and higher in the light | significant and higher in the dark | significant with directional change |
| --- | --- | --- | --- | --- | --- | --- | --- | --- |
| 'BM[c] --> #' | 17.00 | 15.66 | 0.12 | 1 | 0 | 0 | 0 | 0 |
| # --> CO2EX[p]//STA[p]' | 600.00 | 50.00 | 3.58 | 1 | 0 | 1 | 0 | 0 |
| 'CO2[cel] --> #' | 37.60 | 81.66 | -1.12 | 1 | 0 | 0 | 1 | 0 |
| '# <--> Pi[v]' | -1694.03 | 27.83 | 5.93 | 1 | 0 | 1 | 0 | 1 |
| '1.238 ACCOA[p] + 0.075 PYR[c] + 0.36 CO2[cel] … --> BM[c] ' | 17.00 | 15.66 | 0.12 | 1 | 0 | 0 | 0 | 0 |
| 'G6P[p] <--> F6P[p]' | -8.46 | 15.51 | -0.87 | 1 | 3E-144 | 0 | 1 | 1 |
| 'F6P[p] + ATP[p] --> FBP[p] + ADP[p]' | 95.30 | 16.99 | 2.49 | 1 | 0 | 1 | 0 | 0 |
| 'FBP[p] --> F6P[p] + Pi[p]' | 273.34 | 2.23 | 6.94 | 1 | 0 | 1 | 0 | 0 |
| 'FBP[p] <--> DHAP[p] + GAP[p]' | -178.04 | 14.77 | 3.59 | 1 | 0 | 1 | 0 | 1 |
| 'DHAP[p] <--> GAP[p]' | -359.84 | 32.51 | 3.47 | 1 | 0 | 1 | 0 | 1 |
| 'GAP[p] + NADP[p] + Pi[p] <--> NADPH[p] + 13bPG[p]' | -994.49 | 39.38 | 4.66 | 1 | 0 | 1 | 0 | 1 |
| '13bPG[p] + ADP[p] <--> ATP[p] + 3PG[p]' | -994.49 | 39.38 | 4.66 | 1 | 0 | 1 | 0 | 1 |
| '3PG[p] <--> 2PG[p]' | 103.95 | 44.24 | 1.23 | 1 | 0 | 1 | 0 | 0 |
| '2PG[p] <--> PEP[p]' | 103.95 | 44.24 | 1.23 | 1 | 0 | 1 | 0 | 0 |
| 'PEP[p] + ADP[p] --> PYR[p] + ATP[p]' | 207.80 | 30.83 | 2.75 | 1 | 0 | 1 | 0 | 0 |
| 'PYR[p] + ATP[p] --> PEP[p] + AMP[p] + Pi[p]' | 89.08 | 0.92 | 6.60 | 1 | 0 | 1 | 0 | 0 |
| 'AMP[p] + ATP[p] --> 2 ADP[p]' | 89.08 | 0.92 | 6.60 | 1 | 0 | 1 | 0 | 0 |
| 'PYR[p] + E2Pr-lip[p] --> E2Pr-acet-lip[p] + CO2[cel]' | 21.04 | 19.39 | 0.12 | 1 | 0 | 0 | 0 | 0 |
| 'E2Pr-acet-lip[p] --> ACCOA[p] + E2Pr-2hyd-lip[p]' | 21.04 | 19.39 | 0.12 | 1 | 0 | 0 | 0 | 0 |
| 'E2Pr-2hyd-lip[p] + NAD[p] --> E2Pr-lip[p] + NADH[p]' | 21.04 | 19.39 | 0.12 | 1 | 0 | 0 | 0 | 0 |
| 'MAL[p] + NADP[p] --> NADPH[p] + CO2[cel] + PYR[p]' | 0.00 | 0.00 | NaN | NaN | NaN | 0 | 0 | 0 |
| 'G6P[p] + NADP[p] --> NADPH[p] + 6PGL[p]' | 0.04 | 7.10 | -7.33 | 1 | 0 | 0 | 1 | 0 |
| '6PGL[p] --> 6PG[p]' | 0.04 | 7.10 | -7.33 | 1 | 0 | 0 | 1 | 0 |
| '6PG[p] + NADP[p] --> NADPH[p] + RU5P[p] + CO2[cel]' | 0.04 | 7.10 | -7.33 | 1 | 0 | 0 | 1 | 0 |
| 'R5P[p] <--> RU5P[p]' | 203.11 | -2.30 | 6.47 | 1 | 0 | 1 | 0 | 1 |
| 'RU5P[p] <--> XU5P[p]' | -413.97 | -10.90 | 5.25 | 1 | 0 | 1 | 0 | 0 |
| 'GAP[p] + S7P[p] <--> E4P[p] + F6P[p]' | 40.82 | 3.68 | 3.47 | 1 | 0 | 1 | 0 | 0 |
| 'E4P[p] + XU5P[p] <--> F6P[p] + GAP[p]' | -210.40 | -4.42 | 5.57 | 1 | 0 | 1 | 0 | 0 |
| 'S7P[p] + GAP[p] <--> R5P[p] + XU5P[p]' | 203.43 | -2.00 | 6.67 | 1 | 0 | 1 | 0 | 1 |
| 'RU5P[p] + ATP[p] --> RBP[p] + ADP[p]' | 617.13 | 15.70 | 5.30 | 1 | 0 | 1 | 0 | 0 |
| 'CO2EX[p] + RBP[p] --> 2 3PG[p]' | 600.00 | 0.00 | Inf | 1 | 0 | 1 | 0 | 0 |
| 'RBP[p] --> 2PGO[p] + 3PG[p]' | 17.13 | 15.70 | 0.13 | 1 | 0 | 0 | 0 | 0 |
| '2PGO[p] --> GLYCO[p] + Pi[p]' | 17.13 | 15.70 | 0.13 | 1 | 0 | 0 | 0 | 0 |
| 'DHAP[p] + E4P[p] --> SBP[p]' | 244.26 | 1.69 | 7.18 | 1 | 0 | 1 | 0 | 0 |
| 'SBP[p] --> S7P[p] + Pi[p]' | 244.26 | 1.69 | 7.18 | 1 | 0 | 1 | 0 | 0 |
| 'GLYCER[p] + ATP[p] --> 3PG[p] + ADP[p]' | 0.07 | 0.02 | 1.82 | 1 | 0 | 1 | 0 | 0 |
| 'MAL[p] + NAD[p] <--> OAA[p] + NADH[p]' | -5.76 | -5.31 | 0.12 | 1 | 0 | 0 | 0 | 0 |
| 'MAL[p] + NADP[p] <--> OAA[p] + NADPH[p]' | -705.05 | -19.02 | 5.21 | 1 | 0 | 1 | 0 | 0 |
| 'G6P[p] <--> aG6P[p]' | 9.01 | 0.23 | 5.26 | 1 | 0 | 1 | 0 | 0 |
| 'aG6P[p] <--> G1P[p]' | 9.01 | 0.23 | 5.26 | 1 | 0 | 1 | 0 | 0 |
| 'G1P[p] + ATP[p] --> ADP-GLC[p] + 2 Pi[p]' | 9.01 | 0.23 | 5.26 | 1 | 0 | 1 | 0 | 0 |
| '2 ADP-GLC[p] --> 2 ADP[p] + STA[p]' | 4.51 | 0.12 | 5.26 | 1 | 0 | 1 | 0 | 0 |
| 'G6P[c] <--> F6P[c]' | -29.67 | 33.94 | -0.19 | 1 | 0 | 0 | 0 | 0 |
| 'F6P[c] + ATP[c] <--> FBP[c] + ADP[c]' | -31.92 | 31.87 | 0.00 | 1 | 0 | 0 | 0 | 0 |
| 'FBP[c] <--> DHAP[c] + GAP[c]' | -31.92 | 31.87 | 0.00 | 1 | 0 | 0 | 0 | 0 |
| 'GAP[c] <--> DHAP[c]' | 94.44 | -12.38 | 2.93 | 1 | 0 | 1 | 0 | 1 |
| 'GAP[c] + NADP[c] --> 3PG[c] + NADPH[c]' | 89.46 | 0.96 | 6.55 | 1 | 0 | 1 | 0 | 0 |
| 'GAP[c] + NAD[c] + Pi[c] <--> 13dPG[c] + NADH[c]' | -215.82 | 43.30 | 2.32 | 1 | 0 | 1 | 0 | 1 |
| '13dPG[c] + ADP[c] <--> ATP[c] + 3PG[c]' | -215.82 | 43.30 | 2.32 | 1 | 0 | 1 | 0 | 1 |
| '3PG[c] <--> 2PG[c]' | -12.33 | 50.75 | -2.04 | 1 | 0 | 0 | 1 | 1 |
| '2PG[c] <--> PEP[c]' | -12.33 | 50.75 | -2.04 | 1 | 0 | 0 | 1 | 1 |
| 'PYR[c] + ATP[c] --> PEP[c] + AMP[c] + Pi[c]' | 455.87 | 1.38 | 8.37 | 1 | 0 | 1 | 0 | 0 |
| 'AMP[c] + ATP[c] --> 2 ADP[c]' | 455.87 | 1.38 | 8.37 | 1 | 0 | 1 | 0 | 0 |
| 'PEP[c] + ADP[c] --> PYR[c] + ATP[c]' | 30.31 | 23.15 | 0.39 | 1 | 1E-247 | 0 | 0 | 0 |
| 'G6P[c] + NADP[c] --> NADPH[c] + 6PGL[c]' | 0.13 | 8.47 | -5.97 | 1 | 0 | 0 | 1 | 0 |
| '6PGL[c] --> 6PG[c]' | 0.13 | 8.47 | -5.97 | 1 | 0 | 0 | 1 | 0 |
| '6PG[c] + NADP[c] --> NADPH[c] + RU5P[c] + CO2[cel]' | 0.13 | 8.47 | -5.97 | 1 | 0 | 0 | 1 | 0 |
| 'RU5P[c] <--> XU5P[c]' | 0.13 | 8.47 | -5.97 | 1 | 0 | 0 | 1 | 0 |
| 'RU5P[c] <--> R5P[c]' | 0.00 | 0.00 | NaN | NaN | NaN | 0 | 0 | 0 |
| 'OAA[c] + ATP[c] --> CO2[cel] + PEP[c] + ADP[c]' | 31.54 | 0.46 | 6.11 | 1 | 0 | 1 | 0 | 0 |
| 'PEP[c] + CO2[cel] --> OAA[c] + Pi[c]' | 416.07 | 30.92 | 3.75 | 1 | 0 | 1 | 0 | 0 |
| 'CIT[c] + ATP[c] --> ACCOA[c] + OAA[c] + ADP[c] + Pi[c]' | 0.00 | 0.00 | NaN | NaN | NaN | 0 | 0 | 0 |
| 'MAL[c] + NAD[c] <--> OAA[c] + NADH[c]' | 192.27 | -0.20 | 9.88 | 1 | 0 | 1 | 0 | 1 |
| 'MALT[c] --> 2 GLC[c]' | 0.00 | 45.96 | -Inf | 1 | 0 | 0 | 1 | 0 |
| 'GLC[c] + ATP[c] --> ADP[c] + G6P[c]' | 0.00 | 91.93 | -Inf | 1 | 0 | 0 | 1 | 0 |
| 'GLYCO[pe] --> GLYOX[pe]' | 17.13 | 15.70 | 0.13 | 1 | 0 | 0 | 0 | 0 |
| 'GLYOX[pe] + NADH[pe] --> GLY[pe] + NAD[pe]' | 17.06 | 15.68 | 0.12 | 1 | 0 | 0 | 0 | 0 |
| 'SER[pe] + GLYOX[pe] --> GLY[pe] + HPYR[pe]' | 0.07 | 0.02 | 1.82 | 1 | 0 | 1 | 0 | 0 |
| 'HPYR[pe] + NADH[pe] --> NAD[pe] + GLYCER[pe]' | 0.07 | 0.02 | 1.82 | 1 | 0 | 1 | 0 | 0 |
| 'MAL[pe] + NAD[pe] <--> OAA[pe] + NADH[pe]' | 17.13 | 15.70 | 0.13 | 1 | 0 | 0 | 0 | 0 |
| 'PYR[m] + E2Pr-lip[m] --> E2Pr-acet-lip[m] + CO2[cel]' | 22.34 | 31.13 | -0.48 | 1 | 0 | 0 | 0 | 0 |
| 'E2Pr-acet-lip[m] --> ACCOA[m] + E2Pr-2hyd-lip[m]' | 22.34 | 31.13 | -0.48 | 1 | 0 | 0 | 0 | 0 |
| 'E2Pr-2hyd-lip[m] + NAD[m] --> E2Pr-lip[m] + NADH[m]' | 22.34 | 31.13 | -0.48 | 1 | 0 | 0 | 0 | 0 |
| 'ACCOA[m] + OAA[m] --> CIT[m]' | 22.34 | 31.13 | -0.48 | 1 | 0 | 0 | 0 | 0 |
| 'CIT[m] <--> ACO[m]' | 22.34 | 31.13 | -0.48 | 1 | 0 | 0 | 0 | 0 |
| 'ACO[m] <--> ICIT[m]' | 22.34 | 31.13 | -0.48 | 1 | 0 | 0 | 0 | 0 |
| 'ICIT[m] + NAD[m] <--> AKG[m] + CO2[cel] + NADH[m]' | 2.91 | 13.23 | -2.18 | 1 | 0 | 0 | 1 | 0 |
| 'ICIT[m] + NADP[m] <--> AKG[m] + CO2[cel] + NADPH[m]' | 19.43 | 17.90 | 0.12 | 1 | 0 | 0 | 0 | 0 |
| 'AKG[m] + NAD[m] --> SUCCCOA[m] + CO2[cel] + NADH[m]' | 1.18 | 11.63 | -3.31 | 1 | 0 | 0 | 1 | 0 |
| 'SUCCCOA[m] + ADP[m] + Pi[m] <--> SUCC[m] + ATP[m]' | 1.18 | 11.63 | -3.31 | 1 | 0 | 0 | 1 | 0 |
| 'SUCC[m] + UQN[m] <--> FUM[m] + UQL[m]' | 1.18 | 11.63 | -3.31 | 1 | 0 | 0 | 1 | 0 |
| 'UQL[m] + NAD[m] <--> UQN[m] + NADH[m]' | 1.18 | 11.63 | -3.31 | 1 | 0 | 0 | 1 | 0 |
| 'FUM[m] --> MAL[m]' | 0.02 | 10.57 | -8.97 | 1 | 0 | 0 | 1 | 0 |
| 'MAL[m] + NAD[m] --> OAA[m] + NADH[m]' | 146.95 | 16.63 | 3.14 | 1 | 0 | 1 | 0 | 0 |
| 'MAL[m] + NAD[m] --> CO2[cel] + NADH[m] + PYR[m]' | 353.63 | 1.99 | 7.47 | 1 | 0 | 1 | 0 | 0 |
| 'GLY[m] + MTHF[m] <--> THF[m] + SER[m]' | 7.54 | 6.91 | 0.13 | 1 | 0 | 0 | 0 | 0 |
| 'GLY[m] + LP[m] <--> CO2[cel] + SADHLP[m]' | 7.54 | 6.91 | 0.13 | 1 | 0 | 0 | 0 | 0 |
| 'SADHLP[m] + THF[m] <--> MTHF[m] + DLP[m]' | 7.54 | 6.91 | 0.13 | 1 | 0 | 0 | 0 | 0 |
| 'DLP[m] + NAD[m] <--> NADH[m] + LP[m]' | 7.54 | 6.91 | 0.13 | 1 | 0 | 0 | 0 | 0 |
| 'GLYCO[p] <--> GLYCO[c]' | 17.13 | 15.70 | 0.13 | 1 | 0 | 0 | 0 | 0 |
| 'GLYCO[c] <--> GLYCO[pe]' | 17.13 | 15.70 | 0.13 | 1 | 0 | 0 | 0 | 0 |
| 'GLY[pe] <--> GLY[c]' | 17.13 | 15.70 | 0.13 | 1 | 0 | 0 | 0 | 0 |
| 'GLY[c] <--> GLY[m]' | 15.09 | 13.82 | 0.13 | 1 | 0 | 0 | 0 | 0 |
| 'SER[m] <--> SER[c]' | 7.54 | 6.91 | 0.13 | 1 | 0 | 0 | 0 | 0 |
| 'SER[c] <--> SER[pe]' | 0.07 | 0.02 | 1.82 | 1 | 0 | 1 | 0 | 0 |
| 'GLYCER[pe] <--> GLYCER[c]' | 0.07 | 0.02 | 1.82 | 1 | 0 | 1 | 0 | 0 |
| 'GLYCER[c] <--> GLYCER[p]' | 0.07 | 0.02 | 1.82 | 1 | 0 | 1 | 0 | 0 |
| 'MALT[p] --> MALT[c]' | 0.00 | 45.96 | -Inf | 1 | 0 | 0 | 1 | 0 |
| 'G6P[p] + Pi[c] <--> G6P[c] + Pi[p]' | -0.59 | -22.84 | -5.26 | 1 | 2E-124 | 0 | 1 | 0 |
| 'XU5P[p] + Pi[c] <--> XU5P[c] + Pi[p]' | -0.13 | -8.47 | -5.97 | 1 | 0 | 0 | 1 | 0 |
| '3PG[p] + Pi[c] <--> 3PG[c] + Pi[p]' | 114.03 | 6.50 | 4.13 | 1 | 0 | 1 | 0 | 0 |
| 'DHAP[c] + Pi[p] <--> DHAP[p] + Pi[c]' | 62.53 | 19.49 | 1.68 | 1 | 9E-145 | 1 | 0 | 0 |
| 'PEP[c] + Pi[p] <--> PEP[p] + Pi[c]' | 28.71 | -1.48 | 4.27 | 1 | 2E-65 | 1 | 0 | 1 |
| 'PYR[p] <--> PYR[c]' | 95.54 | 8.55 | 3.48 | 1 | 0 | 1 | 0 | 0 |
| 'PYR[c] <--> PYR[m]' | -331.30 | 29.15 | 3.51 | 1 | 0 | 1 | 0 | 1 |
| 'MAL[c] --> MAL[pe]' | 17.13 | 15.70 | 0.13 | 1 | 0 | 0 | 0 | 0 |
| 'OAA[pe] --> OAA[c]' | 17.13 | 15.70 | 0.13 | 1 | 0 | 0 | 0 | 0 |
| 'MAL[c] + Pi[m] <--> MAL[m] + Pi[c]' | 375.95 | 22.55 | 4.06 | 1 | 0 | 1 | 0 | 0 |
| 'OAA[c] + MAL[m] <--> MAL[c] + OAA[m]' | -124.61 | 14.51 | 3.10 | 1 | 0 | 1 | 0 | 1 |
| 'OAA[c] + MAL[p] <--> MAL[c] + OAA[p]' | 710.81 | 24.33 | 4.87 | 1 | 0 | 1 | 0 | 0 |
| 'OAA[c] + CIT[m] <--> CIT[c] + OAA[m]' | 0.00 | 0.00 | NaN | NaN | NaN | 0 | 0 | 0 |
| 'Pi[c] --> Pi[m]' | 1626.19 | 223.96 | 2.86 | 1 | 0 | 1 | 0 | 0 |
| 'Pi[c] <--> Pi[p]' | -1794.75 | -64.48 | 4.80 | 1 | 0 | 1 | 0 | 0 |
| 'Pi[v] <--> Pi[c]' | -1694.03 | 27.83 | 5.93 | 1 | 0 | 1 | 0 | 1 |
| 'ATP[m] + ADP[c] --> ADP[m] + ATP[c]' | 1240.95 | 192.84 | 2.69 | 1 | 0 | 1 | 0 | 0 |
| 'ATP[p] + ADP[c] <--> ADP[p] + ATP[c]' | -41.87 | -38.08 | 0.14 | 0 | 0.08 | 0 | 0 | 0 |
| '2 Hv[p] + PQN[p] --> PQL[p] + 2 H_nc[p]' | 1736.94 | 0.00 | Inf | 1 | 0 | 1 | 0 | 0 |
| 'PQL[p] + 2 PC_o[p] <--> PQN[p] + 2 PC_r[p] + 2 H_nc[p]' | 1736.94 | 0.00 | Inf | 1 | 0 | 1 | 0 | 0 |
| 'Hv[p] + PC_r[p] + FE_o[p] --> PC_o[p] + FE_r[p]' | 3473.87 | 0.00 | Inf | 1 | 0 | 1 | 0 | 0 |
| '2 FE_r[p] + NADP[p] --> 2 FE_o[p] + NADPH[p] + 2 H_nc[p]' | 1736.94 | 0.00 | Inf | 1 | 0 | 1 | 0 | 0 |
| 'Hv[p] --> 2 H_c[p]' | 868.47 | 0.00 | Inf | 1 | 0 | 1 | 0 | 0 |
| '# --> Hv[p]' | 7816.21 | 0.00 | Inf | 1 | 0 | 1 | 0 | 0 |
| '2 H_c[p] + 12 H_nc[p] + 3 ADP[p] --> 3 ATP[p]' | 868.47 | 0.00 | Inf | 1 | 0 | 1 | 0 | 0 |
| 'ATP[p] --> ADP[p] + ATP_maint[cel]' | 881.86 | 0.64 | 10.42 | 1 | 0 | 1 | 0 | 0 |
| '2.4 ADP[m] + NADH[m] + 2.4 Pi[m] --> NAD[m] + 2.4 ATP[m]' | 535.73 | 93.16 | 2.52 | 1 | 0 | 1 | 0 | 0 |
| 'ATP_maint[cel] --> #' | 881.86 | 0.64 | 10.42 | 1 | 0 | 1 | 0 | 0 |
| 'NADP[c] + NADH[c] <--> NADPH[c] + NAD[c]' | -13.03 | 52.79 | -2.02 | 1 | 0 | 0 | 1 | 1 |
|  |  |  |  |  | **SUM:** | **66** | **23** | **19** |

**Table S8:**

*Overview on the different ratios between non cyclic and cyclic electron flow that were tested for, and their corresponding ATP:NADPH ratios.*

| number of electrons in non-cyclic photosynthesis | number of electrons in cyclic photosynthesis | ATP produced | NADPH produced | ATP:NADPH ratio |
| --- | --- | --- | --- | --- |
| 14 | 0 | 3 | 2.333 | 1.285898 |
| 13 | 1 | 6 | 4.333 | 1.384722 |
| 12 | 2 | 3 | 2 | 1.5 |
| 11 | 3 | 3 | 1.833 | 1.636661 |
| 10 | 4 | 3 | 1.667 | 1.79964 |
| 9 | 5 | 3 | 1.5 | 2 |
| 8 | 6 | 3 | 1.333 | 2.250563 |
| 7 | 7 | 3 | 1.167 | 2.570694 |
| 6 | 8 | 3 | 1 | 3 |
| 5 | 9 | 3.6 | 1 | 3.6 |
| 4 | 10 | 4.5 | 1 | 4.5 |
| 3 | 11 | 6 | 1 | 6 |
| 2 | 12 | 9 | 1 | 9 |
| 1 | 13 | 18 | 1 | 18 |
| 0 | 14 | 3 | 0 | NaN |


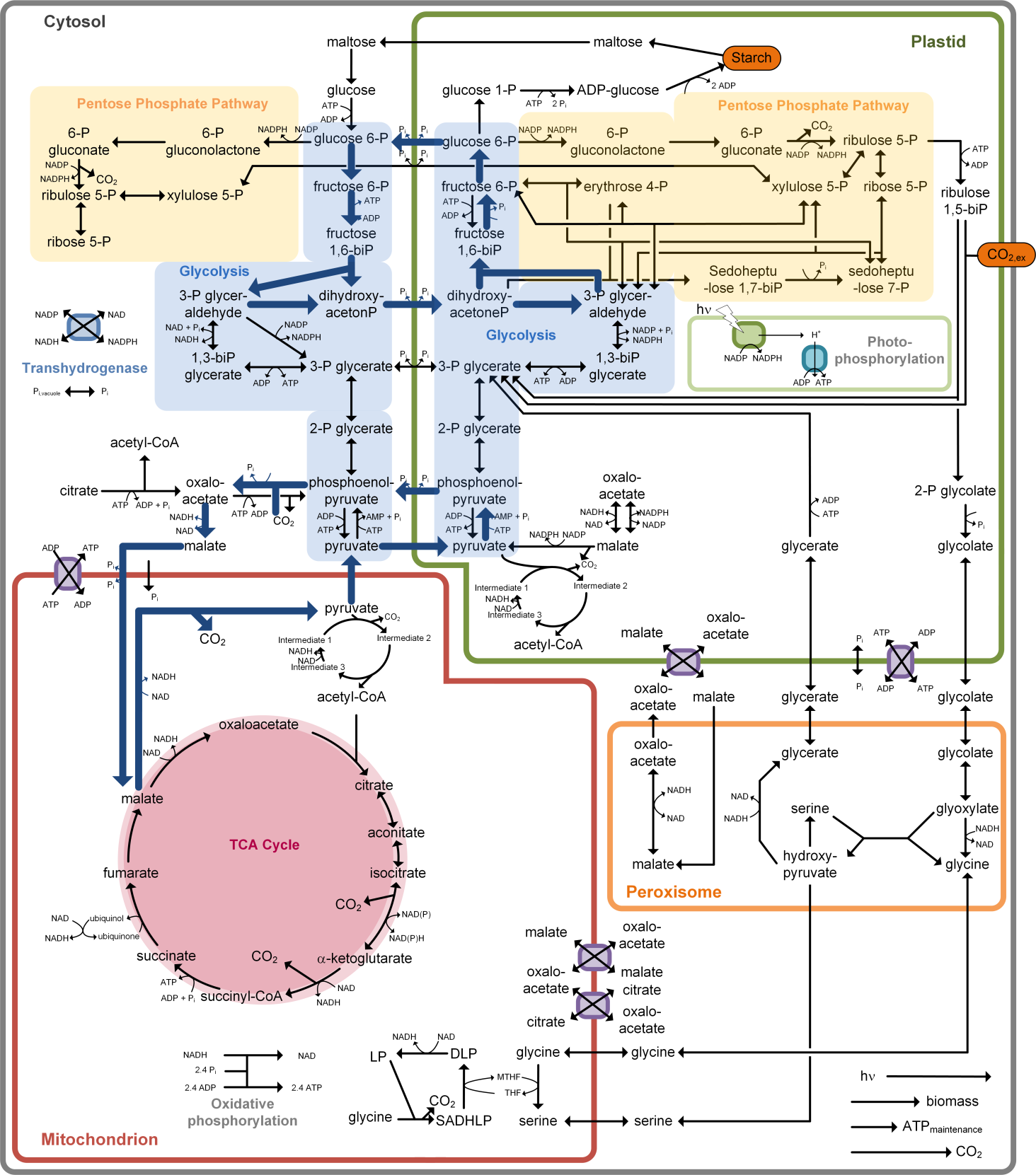


**Figure S1:** Two exemplified futile cycles in the proposed metabolic network causing energetic inefficiency. The futile cycle across the mitochondrial membrane consumes one plastidic ATP per cycle, whereas the futile cycle involving the two copies of the EMP pathway requires one cytosolic ATP per cycle.

**
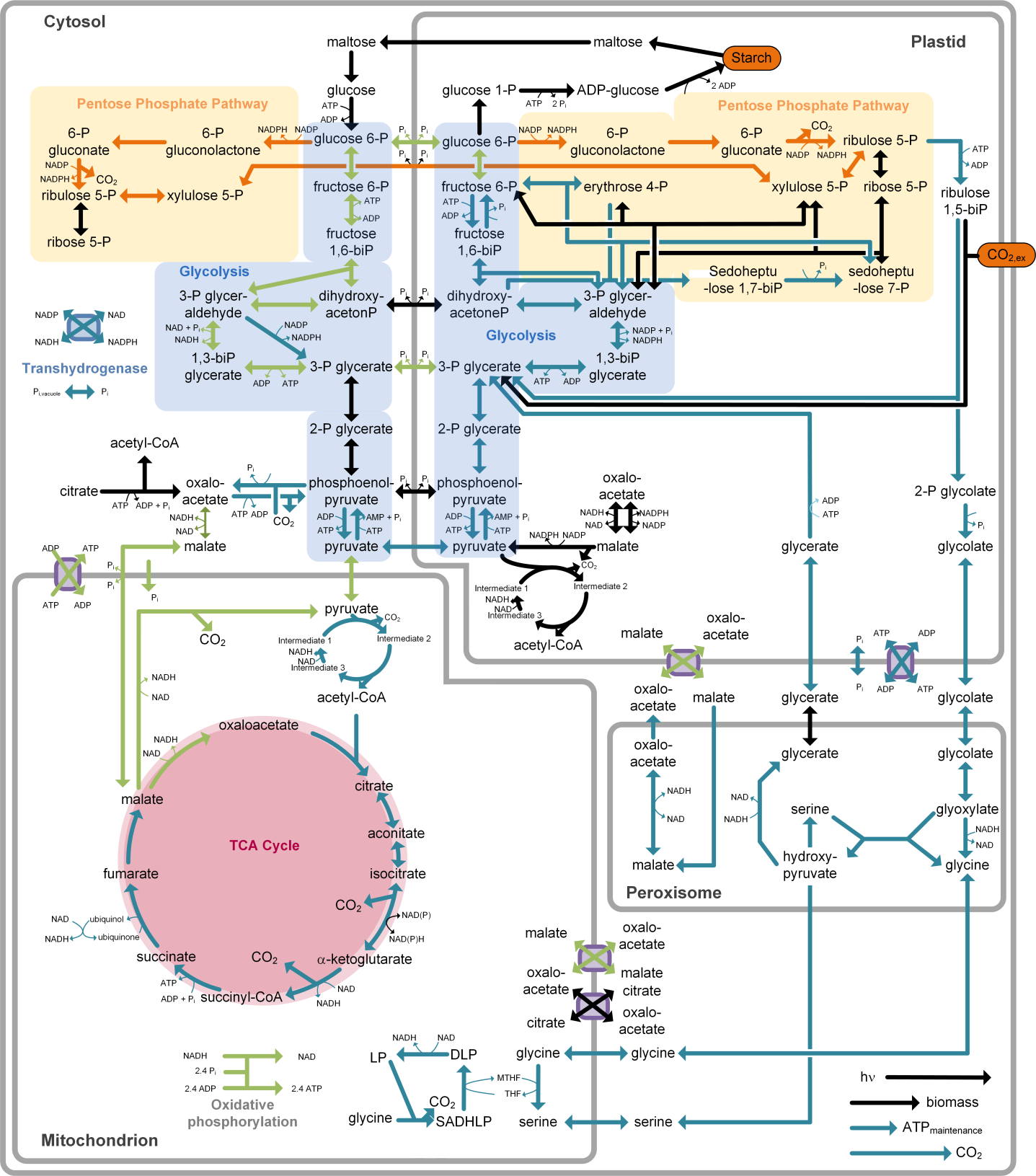
**

**Figure S2:** Flux change for increasing contribution of cyclic electron flow to photophosphorylation. Blue arrows represent reactions that display a change in flux for a high contribution of cyclic electron flow, but remain constant for lower contributions. Green arrows represent fluxes that are increased for increasing non-cyclic contribution to photophosphorylation. Red arrows display a stable flux profile with a distinct maximum around 12:2. Black fluxes remain unchanged throughout the different tested ratios.

**
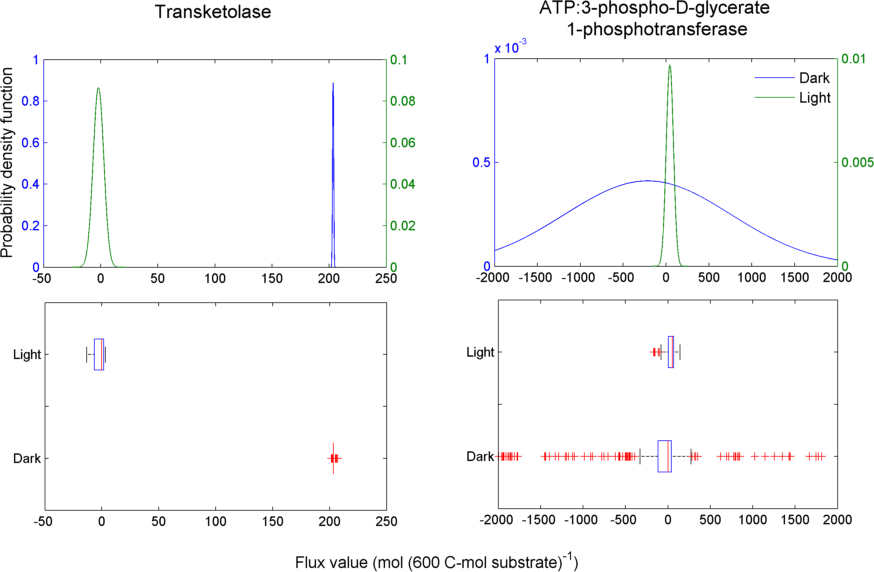
**

**Figure S3:** Exemplified analysis of the optimal (top 1%) biomass producing fluxes in the light and the dark. Both transketolase and ATP: 3-phospho-D-glycerate 1-phosphotransferase display a significant change in flux between dark and light with a reversal of flux direction (Supplemental Table S7). The upper panels show the normal probability density function for the light and dark metabolism of both enzymes based on the mean and standard deviation of the top 1% modes. The bottom panels display the boxplots of the respective data. From this figure it becomes clear that individual alternative solutions exist, in which no flux reversal occurs.

**Figure S4:** Detailed exemplary network to assess the validity of our network condensation. The network describes metabolic conversion from a substrate A into biomass components G and J. Linear pathways exist between metabolites C/D/E and biomass components G and J. Furthermore, two parallel pathways for the conversion of metabolite A to C exist.

**Figure S5:** Exemplary network from Figure S4 that describes the metabolic conversion from a substrate A into biomass components G and J. In this model, the linear pathways between metabolites C/D/E and biomass components G and J are lumped together. However, the two parallel pathways for the conversion of metabolite A to C remain.

**Figure S6:** Exemplary network from Figure S4 that describes the metabolic conversion of a substrate A into biomass components G and J. In this model, the linear pathways between metabolites C/D/E and biomass components G and J are lumped. In addition, the two parallel pathways for the conversion of metabolite A to C have been lumped into one metabolic conversion.

**Additional information S1:**

The following simulations demonstrate the validity of the performed model condensation and confirm that the condensed model is descriptive of the entire set of metabolic conversions.

Let us assume an exemplary network (Figure S4), which describes metabolic conversion of a substrate A into biomass components G and J. Two parallel pathways for the conversion of metabolite A to C exist. This detailed network allows for the computation of 8 different elementary flux modes (Table S6).

Table S6: Elementary flux modes of the detailed network depicted in Figure S4.

|  | EFM1 | EFM2 | EFM3 | EFM4 | EFM5 | EFM6 | EFM7 | EFM8 |
| --- | --- | --- | --- | --- | --- | --- | --- | --- |
| ' --> A' | 1 | 1 | 1 | 1 | 2 | 2 | 2 | 2 |
| 'A --> B' | 1 | 0 | 1 | 0 | 2 | 0 | 2 | 0 |
| 'B --> C' | 0 | 0 | 1 | 1 | 2 | 2 | 0 | 0 |
| 'C --> D' | 1 | 1 | 1 | 1 | 1 | 1 | 1 | 1 |
| 'D --> E' | 1 | 1 | 1 | 1 | 0 | 0 | 0 | 0 |
| 'D --> F' | 0 | 0 | 0 | 0 | 1 | 1 | 1 | 1 |
| 'C + F --> G' | 0 | 0 | 0 | 0 | 1 | 1 | 1 | 1 |
| 'G --> BMC' | 0 | 0 | 0 | 0 | 1 | 1 | 1 | 1 |
| 'E --> H' | 1 | 1 | 1 | 1 | 0 | 0 | 0 | 0 |
| 'H--> J' | 1 | 1 | 1 | 1 | 0 | 0 | 0 | 0 |
| 'J --> BMC' | 1 | 1 | 1 | 1 | 0 | 0 | 0 | 0 |
| 'Z <==> B' | -1 | 0 | 0 | 1 | 0 | 2 | -2 | 0 |
| 'A --> Z' | 0 | 1 | 0 | 1 | 0 | 2 | 0 | 2 |
| 'Z --> C' | 1 | 1 | 0 | 0 | 0 | 0 | 2 | 2 |
| 'BMC --> ' | 1 | 1 | 1 | 1 | 1 | 1 | 1 | 1 |

As most of the peripheric biosynthetic pathways are linear and metabolic steady-state is given, they can easily be summarized into a single, lumped biomass equation, starting from central metabolic precursor metabolites, without noteworthy degeneration of information content. In the detailed exemplary model, this means that the 3 conversions between metabolite E and biomass (BMC) can be reduced to one reaction, where E goes to BMC (Figure S5). Similarly, the reactions between C, D and BMC can be summarized as C + D deliver BMC. It is demonstrated that no elementary mode information is lost through reduction of linear pathways, as the same 8 elementary flux modes are found for the network with lumped linear pathways (Table S7).

Table S7: Elementary flux modes of the simplified network depicted in Figure S5.

|  | EFM1 | EFM2 | EFM3 | EFM4 | EFM5 | EFM6 | EFM7 | EFM8 |
| --- | --- | --- | --- | --- | --- | --- | --- | --- |
| ' --> A' | 1 | 1 | 1 | 1 | 2 | 2 | 2 | 2 |
| 'A --> B' | 1 | 0 | 1 | 0 | 2 | 0 | 2 | 0 |
| 'B --> C' | 0 | 0 | 1 | 1 | 2 | 2 | 0 | 0 |
| 'C --> D' | 1 | 1 | 1 | 1 | 1 | 1 | 1 | 1 |
| 'D --> E' | 1 | 1 | 1 | 1 | 0 | 0 | 0 | 0 |
| 'C + D --> BMC' | 0 | 0 | 0 | 0 | 1 | 1 | 1 | 1 |
| 'E --> BMC' | 1 | 1 | 1 | 1 | 0 | 0 | 0 | 0 |
| 'Z <==> B' | -1 | 0 | 0 | 1 | 0 | 2 | -2 | 0 |
| 'A --> Z' | 0 | 1 | 0 | 1 | 0 | 2 | 0 | 2 |
| 'Z --> C' | 1 | 1 | 0 | 0 | 0 | 0 | 2 | 2 |
| 'BMC --> ' | 1 | 1 | 1 | 1 | 1 | 1 | 1 | 1 |

The second way to reduce network size, involved merging of parallel pathways. In the exemplary network two parallel pathways exist between metabolites A and C that can be summarized (Fig. S6). In this case, there is a loss in information, as it is now impossible to distinguish between the two parallel pathways. However, the observed loss in information doesn’t influence the overall behavior of the metabolic network. The elementary flux modes in table S6 can be grouped into EFM1-4 with a net flux from A to C of 1 and EFM5-8 with a net flux of 2. Both groups only differ in the usage of the parallel pathways between A and C, all remaining flux values are identical. So if the network would be simplified with a merger of the parallel pathways between metabolites A and C, it is expected that only two flux modes remain that still describe the metabolic capabilities inherent to the model and only information is lost on the usage of the parallel pathways. Indeed, the network depicted in figure S6 is described by the two previously mentioned flux modes (Table S8) and thus, still describes the entire solution space of the network.

Table S8: Elementary flux modes of the simplified network depicted in Figure S6.

|  | EFM1 | EFM2 |
| --- | --- | --- |
| ' --> A' | 1 | 2 |
| 'A --> Y' | 1 | 2 |
| 'Y --> C' | 1 | 2 |
| 'C --> D' | 1 | 1 |
| 'D --> E' | 1 | 0 |
| 'C + D --> BMC' | 0 | 1 |
| 'E --> BMC' | 1 | 0 |
| 'BMC --> ' | 1 | 1 |

In addition, simulations were performed with an *A. thaliana* model to show that no relevant information is lost by the condensation. Two additional scenarios were computed.

Table S9: Additions to the metabolic network for computation of linear and parallel pathways. Additional abbreviations: ACLA: (S)-2-Acetolactate, HMBUT: 3-Hydroxy-3-methyl-2-oxobutanoicacid, DHMBUT: (R)-2,3-Dihydroxy-3-methylbutanoate, MOBUT: 3-Methyl-2-oxobutanoicacid, IPM: alpha-Isopropylmalate, 2IPM: 2-Isopropylmaleate, 3IPM: (2R,3S)-3-Isopropylmalate, 2IOS: (2S)-2-Isopropyl-3-oxosuccinate, MOP: 4-Methyl-2-oxopentanoate, LEU: L-Leucine, GLUT: L-Glutamate.

|  | Linear pathway of leucine biosynthesis | Parallel pathways of glutamate biosynthesis |
| --- | --- | --- |
| Replacement of biomass equation | '(1.232) ACCOA[p] + (0.075) PYR[c] + (0.361) CO2[cel] + (0.820) PEP[p] + (1.245) AKG[m] + (0.019) R5P[p] + (0.004) DHAP[p] + (0.410) E4P[p] + (0.455) OAA[c] + 0.278 3PG[p] + (0.115) PYR[p] + (2.195) NADPH[p] + (4.513) NADPH[c] + (1.143) NADPH[m] + (0.905) NADH[p] + (0.619) NAD[c] + (0.114) GAP[p] + (0.132) F6P[c] + (1.703) G6P[c] + (0.2652) STA[p] + (0.068) FUM[m] + (0.050) MAL[c] + 0.440 SER[c] + 0.120 GLY[c] + (4.652) ATP[p] + (6.013) ATP[c] + (2.705) ATP[m] + (0.0055) LEU[p] --> BM[c] + (2.195) NADP[p] + (4.513) NADP[c] + (1.143) NADP[m] + (0.905) NAD[p] + (0.619) NADH[c] + (4.652) ADP[p] + (6.013) ADP[c] + (2.705) ADP[m] + (4.933) Pi[c] + (8.023) Pi[p] + (2.158) Pi[m]' | '(1.238) ACCOA[p] + (0.075) PYR[c] + (0.360) CO2[cel] + (0.820) PEP[p] + (0.6493) AKG[m] + (0.019) R5P[p] + (0.004) DHAP[p] + (0.410) E4P[p] + (0.455) OAA[c] + 0.278 3PG[p] + (0.126) PYR[p] + (2.206) NADPH[p] + (4.513) NADPH[c] + (0.5473) NADPH[m] + (0.899) NADH[p] + (0.619) NAD[c] + (0.114) GAP[p] + (0.132) F6P[c] + (1.703) G6P[c] + (0.2652) STA[p] + (0.068) FUM[m] + (0.050) MAL[c] + 0.440 SER[c] + 0.120 GLY[c] + (4.652) ATP[p] + (6.013) ATP[c] + (2.705) ATP[m] + (0.5957) GLUT[m] --> BM[c] + (2.206) NADP[p] + (4.513) NADP[c] + (0.5473) NADP[m] + (0.899) NAD[p] + (0.619) NADH[c] + (4.652) ADP[p] + (6.013) ADP[c] + (2.705) ADP[m] + (4.933) Pi[c] + (8.023) Pi[p] + (2.158) Pi[m]' |
| Additions to the network | '2 PYR[p] --> CO2[cel] + ACLA[p]' | 'GLUT[m] + NAD[m] <==> AKG[m] + NADH[m]' |
|  | 'ACLA[p] <==> HMBUT[p]' | 'GLUT[m] + NADP[m] <==> AKG[m] + NADPH[m]' |
|  | 'HMBUT[p] + NADPH[p] <==> NADP[p] + DHMBUT[p]' |  |
|  | 'DHMBUT[p] --> MOBUT[p]' |  |
|  | 'MOBUT[p] + ACCOA[p] --> IPM[p]' |  |
|  | 'IPM[p] <==> 2IPM[p]' |  |
|  | '2IPM[p] <==> 3IPM[p]' |  |
|  | '3IPM[p] + NAD[p] <==> NADH[p] + 2IOS[p]' |  |
|  | '2IOS[p] --> CO2[cel] + MOP[p]' |  |
|  | 'MOP[p] + GLUT[p] --> AKG[p] + LEU[p]' |  |
|  | 'GLUT[p] + NADP[p] <==> AKG[p] + NADPH[p]' |  |

In the first model, the linear pathway of leucine biosynthesis was excluded from the lumped biomass equation and the individual reactions added to the condensed network model (Table S9).

In the second model, glutamate biosynthesis was excluded from the lumped biomass equation and the two parallel pathways between the glutamate precursor, alpha-ketoglutarate, and glutamate where added to the network (Table S9).

Next the elementary modes for the two models were calculated for autotrophic conditions and the number of modes, the maximum theoretical biomass yield, as well the time and memory required for the enumeration were assessed (Table S10).

Table S10: Comparison of network parameters and computation requirements for the condensed metabolic network and two extensions hereof, describing the linear pathways for leucine biosynthesis and the parallel pathways for glutamate biosynthesis, respectively.

|  | **condensed** | **linear** | **parallel** |
| --- | --- | --- | --- |
| **time (s)** | 124 | 138 | 590 |
| **maxY  (*g DW (mol C)^-1^)*** | 28.3 | 28.3 | 28.3 |
| **# modes** | 1 206 894 | 1 206 894 | 4 628 926 |
| **RAM (GB)** | 2.7 | 2.7 | 3.8 |

From Table S10 it can be discerned that lumping linear pathways does not influence the calculated elementary flux mode solution space, as both the number of modes and the maximal biomass yield remain unaltered. However, an increase in computation time is associated with a larger set of reactions. In addition, it becomes obvious that calculation of parallel pathways dramatically increases both computation time and memory requirement. The latter, is undoubtedly contributed to the increase in calculated flux modes. However, the general behavior of the model is unchanged, as the calculated yield is unaffected. From the simulations with the exemplary network model and the extended condensed model for *A. thaliana*, it can be concluded that condensation of a metabolic model in the described fashion, does not influence the overall behavior of the system and no relevant information is lost.
